# Neisseria Heparin Binding Antigen is targeted by the human alternative pathway C3-convertase

**DOI:** 10.1371/journal.pone.0194662

**Published:** 2018-03-26

**Authors:** Martina Di Fede, Massimiliano Biagini, Elena Cartocci, Carlo Parillo, Alessandra Greco, Manuele Martinelli, Sara Marchi, Alfredo Pezzicoli, Isabel Delany, Silvia Rossi Paccani

**Affiliations:** GSK, Siena, Italy; Universidad Nacional de la Plata, ARGENTINA

## Abstract

Neisserial Heparin Binding Antigen (NHBA) is a surface-exposed lipoprotein specific for *Neisseria* and constitutes one of the three main protein antigens of the Bexsero vaccine. Meningococcal and human proteases, cleave NHBA protein upstream or downstream of a conserved Arg-rich region, respectively. The cleavage results in the release of the C-terminal portion of the protein. The C-terminal fragment originating from the processing of meningococcal proteases, referred to as C2 fragment, exerts a toxic effect on endothelial cells altering the endothelial permeability. In this work, we reported that recombinant C2 fragment has no influence on the integrity of human airway epithelial cell monolayers, consistent with previous findings showing that *Neisseria meningitidis* traverses the epithelial barrier without disrupting the junctional structures. We showed that epithelial cells constantly secrete proteases responsible for a rapid processing of C2 fragment, generating a new fragment that does not contain the Arg-rich region, a putative docking domain reported to be essential for C2-mediated toxic effect. Moreover, we found that the C3-convertase of the alternative complement pathway is one of the proteases responsible for this processing. Overall, our data provide new insights on the cleavage of NHBA protein during meningococcal infection. NHBA cleavage may occur at different stages of the infection, and it likely has a different role depending on the environment the bacterium is interacting with.

## Introduction

*Neisseria meningitidis*, a gram-negative obligate human commensal typically residing in nasopharyngeal mucosa, is a pathogenic member of the *Neisseria* family and a leading cause of fatal sepsis and bacterial meningitis worldwide. Based on the immunologic reactivity of the capsular polysaccharides, 12 distinct serogroups have been defined (A, B, C, E, H, I, K, L, W, X, Y and Z), six of which cause life-threatening disease (A, B, C, W, X and Y) [**1**]. In non-epidemic settings, approximately 10% of healthy individuals at any time carry *N*. *meningitidis* in the upper airways [**2**]. Acquisition of the bacteria from a healthy carrier or an infected person occurs through close direct contact with respiratory droplets or secretions.

Colonization of human nasopharyngeal mucosa by *N*. *meningitidis* is the first step in establishment of both carrier state and invasive meningococcal disease. Bacteria attach selectively to and enter non-ciliated columnar cells of the nasopharyngeal mucosa [**3**]. After adhesion, interaction of *N*. *meningitidis* with epithelial cell induces the reorganization of the cell surface. Microvilli of epithelial cells elongate surrounding the bacteria and eventually engulf meningococci by invagination of the cell membrane and vacuole formation [**3**]. Penetration of meningococci through the epithelial layer occurs by a transcellular route without disrupting the epithelium integrity [**4**; **5**], and this is not an unusual event since bacteria are found in sub-epithelial tissue of healthy individuals [**6**]. While in asymptomatic carriers, bacteria that cross the epithelial barrier are eliminated; in susceptible individuals, bacteria survive and enter the bloodstream resulting in a systemic infection. Frequently, meningococci translocate from the bloodstream across the blood-brain barrier, proliferate in the cerebrospinal fluid, and cause meningitis.

Neisseria Heparin Binding Antigen (NHBA), also known as GNA2132 (Genome-derived Neisseria Antigen 2132), is a surface-exposed lipoprotein specific for *Neisseria*, and it is one of the three main protein antigens of the Bexsero vaccine against *N*. *meningitidis* serogroup B. NHBA has been implicated in different steps of meningococcal pathogenesis, including bacterial adhesion to epithelial cells, biofilm formation, bacterial survival in the blood and vascular leakage [**7**; **8**; **9**; **10**]. NHBA protein can be structurally divided into an N-terminal and a C-terminal domain separated by a conserved Arginine (Arg)-rich region, which is responsible for the protein binding to heparin and heparan sulfate proteoglycans and it is the target of several proteases [**7**; **10**]. The proteolytic processing of NHBA protein results in the release of its C-terminal portion. Human proteases, including lactoferrin and kallikrein, cleave the protein downstream the Arg-rich region releasing a C-terminal fragment that does not contain the Arg-rich region [**7**; **11**]. In a subset of *N*. *meningitidis* hypervirulent strains, meningococcal NalP protease cleaves NHBA protein upstream the Arg-rich region, generating a C-terminal fragment that contains the Arg-rich region; this fragment is referred to as C2 fragment [**7**]. Recombinant C2 fragment has been shown to enter endothelial cells and to alter the endothelial permeability *in vitro*. Reactive oxygen species production and phosphorylation/degradation of the adherens-junction protein VE-cadherin are involved in this latter process [**9]**.

Since NHBA expression is upregulated at 32°C [**12**], a temperature encountered during *N*. *meningitidis* initial colonization of the upper respiratory tract [**13**], we hypothesized that the C2 fragment could also alter the epithelial permeability and facilitate the traversal of *N*. *meningitidis* through the epithelium. To this end, we verified the effect of the C2 fragment on polarized Calu-3 cells, a cell line resembling the morphological features of a differentiated human airway epithelium. In line with previous studies showing that *N*. *meningitidis* traverses the epithelial barrier without disrupting the junctional structures [**3**; **4**; **5**], we observed no influence of C2 fragment on the cellular integrity. Unexpectedly, we found out that epithelial cells were able to process both C2 fragment and NHBA full-length protein. We identified the cleavage site of epithelial cell proteases within NHBA protein and finally we verified that the C3-convertase of the alternative complement pathway is one of the proteases responsible for this cleavage.

## Materials and methods

### Cell culture

Calu-3 epithelial cells, derived from lung adenocarcinoma (HTB-55; ATCC), were cultured in T75 flasks, with DMEM:F12 supplemented with L-Glutamine (Gibco-Themo Fischer Scientific), 10% (v/v) fetal bovine serum (Gibco-Themo Fischer Scientific) and Penicillin-Streptomycin (Gibco-Themo Fischer Scientific), at 37°C in 5% CO_2_. Polarization of Calu-3 cells was performed according to the method developed by Sutherland et al. [**5]**. Briefly, cell monolayers were grown on 0.33 cm^2^, 1μm-pore-size, BD Falcon cell culture inserts (BD Bioscience) containing polyethylene terephthalate membranes in 24-well plate. Calu-3 cells, between passages 4 and 8, were seeded at a density of 0.15 × 10^6^ cells per transwell, onto the apical side of membranes that were previously coated with a solution of collagen type I from rat tail (10μg/cm^2^; Sigma). Cell monolayers were maintained with 0.8 mL of culture medium in the apical chamber and 0.5 mL in the basolateral chamber. Cells were allowed to grow and differentiate for 5–6 days, with the media changed every second day. Cell polarity and tight junction barrier function were verified by measuring trans-epithelial electrical resistance (TEER) using an epithelial voltohmmeter (EVOM2; World Precision Instruments) attached to STX chopstick electrodes (World Precision Instruments). Cultures with TEER values of more than 1,400 Ω were retained for experimentation. On the previous day of each experiment performed with Calu-3 cells, or polarized Calu-3 cells, or cell derivate of these cells, serum concentration in the culture medium was decreased to 1% (v/v). On the experimental day, cell monolayers were abundantly washed with DMEM:F12 supplemented with L-Glutamine (Gibco-Themo Fischer Scientific), from now referred as DMEM/F12 medium, in order to eliminate any trace of serum prior to perform the experiment.

Primary normal human bronchial epithelial (NHBE) cells, isolated from a single healthy donor (Lonza CC-2540S), were differentiated according to the manufacturer’s instructions. Briefly, cells were expanded in T75 flasks, using bronchial epithelial basal growth medium (BEBM; Lonza) supplemented with the BEGM (bronchial epithelial cell growth medium) BulletKit (Lonza), as recommended by the supplier, at 37°C in 5% CO_2_ until ~80% confluence and used between passages 1 and 3. Then, cells were dissociated using StemPro Accutase cell dissociation reagent (Life Technologies) and were seeded onto semipermeable membrane supports (12-mm diameter, 0.4-mpore size; Costar) that were previously coated with a solution of collagen type I from rat tail (Gibco) at a concentration of 0.03 mg/mL. Cells were seeded at a density of 0.1 x 10^6^ cells per well using bronchial air-liquid interface (B-ALI) medium (Lonza) supplemented with the B-ALI BulletKit (Lonza). When confluence was reached, the apical medium was removed, and an air-liquid interface was established to trigger differentiation. Cells were maintained at the ALI for at least 28 days prior to use in biological assays, being the basolateral medium changed every second day, to ensure a differentiated cell population with a mucociliary phenotype. The apical side was rinsed with phosphate-buffered saline (PBS) every week to remove excess mucus production. Cell polarity and tight junction barrier function were verified by measuring trans-epithelial electrical resistance (TEER) using an epithelial volt ohmmeter (EVOM2; World Precision Instruments) attached to STX chopstick electrodes (World Precision Instruments). Cultures with TEER values of more than 1,200 Ω were retained for experimentation.

### Cell supernatant preparation

Cell supernatants were recovered from Calu-3, or polarized Calu-3, or differentiated NHBE cells. For Calu-3 and polarized Calu-3 cells, cell supernatants were recovered after incubation of cells in DMEM/F12 medium during at least 8h or overnight, at 37°C and 5% CO_2_. Cell supernatant were then collected and stored at 4°C or -20°C until use. For differentiated NHBE cells, PBS washes of the apical side routinely performed for removing excess of mucus were collected and stored at 4°C or -20°C until use.

### Bacterial strains, growth conditions and working seed preparation

*N*. *meningitidis* strain M2934 is a clinical isolate from the United States of America [**14]**, and it belongs to cc32 (ST-32) **[15]**. M2934 strain was cultured on GC agar plates (Difco) or DMEM/F12 medium at 37 °C plus 5% CO_2_. Working seeds were prepared inoculating overnight colonies grown on GC agar plate in 25ml of DMEM/F12 medium contained in 125mL- Corning Erlenmeyer cell culture flask (Sigma). OD_600nm_ ~ 0.1 was used as start culture density. Liquid culture was grown at 37 °C plus 5% CO_2_, under aerobic condition (180 rpm) until OD_600nm_ ~ 1. After adding glycerol (final concentration of 15% (v/v)), liquid culture was aliquoted in 1mL criovials and stored at -80 °C until use. For cleavage assay on live bacteria, 4 working seeds (OD_600nm_ ~ 0.6) were used to inoculate ~20 ml DMEM/F12 medium contained in 125mL- Corning Erlenmeyer cell culture flasks (Sigma). Liquid cultures were grown at 37 °C plus 5% CO_2_, under aerobic condition (180 rpm). OD_600nm_ ~ 0.1 was used as start culture density. Liquid culture was grown at 37 °C plus 5% CO_2_, under aerobic condition (180 rpm) until OD_600nm_ ~ 0.5, and then used for experimentation.

*E*. *coli* strains BL21 (DE3) (Invitrogen) were cultured at 37 °C in Luria Bertani (LB) agar plates, or LB broth, or using EnPresso B growth kit (Biosilta); when required ampicillin was added in the medium at a final concentrations of 100 μg/mL. Liquid cultures prepared with LB broth were grown at 37 °C, under aerobic condition (180 rpm); while liquid cultures prepared using EnPresso B medium were grown according to the manufacturer’s instructions. Details of the strains used in this work are reported in S1 Text.

### SDS-PAGE and Western blot analysis

Proteins were separated by SDS-PAGE electrophoresis using 4–12% or 12% polyacrylamide NuPAGE Bis-Tris Precast Gels (Invitrogen). For SDS-PAGE analysis, gels were stained with SimplyBlue Safe Stain (Invitrogen). For Western blot (WB) analysis, proteins contained in the gels were transferred onto nitrocellulose membranes. Western blots were performed according to standard procedures. NHBA full-length protein and its C-terminal fragments were identified with polyclonal mouse antisera raised against the recombinant NHBA full-length protein (working dilution 1:1,000) or against the recombinant C2 fragment (working dilution 1:1,000), respectively. An anti-mouse antiserum conjugated to horseradish peroxidase (Dako) was used as secondary antibody. Bands were visualized with Super Signal West Pico Chemiluminescent Substrate (Pierce) following the manufacturer’s instructions. Densitometric analysis of protein bands was performed using Image J software.

### NHBA cleavage assay using recombinant proteins

Both recombinant C2 fragment and NHBA full-length protein was used as substrates for the assay. Cell monolayers, cell supernatants, fractions of cell supernatants, plasma-purified kallikrein (Sigma), human sera (Complement Technology), purified complement components (Calbiochem) were used as protease sources for the assay. 0.5 μM or 5μM of recombinant protein was incubated at 37 °C with protease source or with DMEM/12 medium, as negative control, for various time intervals (45 min, 1 h, 2 h, 4 h, 24 h). For purified complement components, C3b (5 μg), factor B (fB, 10 μg), and properdin (P, 1 μg) were incubated for 35 min at room temperature in 20 mM Hepes (pH 7.5), 75 mM NaCl, and 5 mM MgCl_2_ at a final molar ratio C3b:fB:P 1:4:0.7. Subsequently, 100 ng of factor D and 1 μg of NHBA protein were added, and the mixture was further incubated for 1 h at 37°C. At each time point, samples were collected and the cleavage of substrates was evaluated by SDS-PAGE or WB analysis. When required, protease sources were pre-treated with protease inhibitors 30 min before adding the substrate. Protease inhibitors used in this work were the followings: EDTA (Sigma), Leupeptin (Sigma), Pepstatin A (Sigma), GI254023X (Sigma), E-64 (Sigma).

#### NHBA cleavage assay using live bacteria

5 mL of cell supernatant or DMEM/12 medium, as negative control, were incubated for 2 h, at 37 °C, 5% CO_2_, with *N*. *meningitidis* strain M2934. For each condition tested, 2 mL of liquid culture of M2934 strain grown until OD_600nm_ ~ 0.5 was used to prepare the inoculum. After 2 h of incubation, samples were centrifuged at 3,500x*g*, for 10 min in order to collect both bacterial pellets and supernatants. Bacteria were immediately processed for FACS analysis. Supernatants were filtered using 0.22 μm filter, concentrated using 10,000 MW Amicon Ultra Centrifugal Filters (Millipore), and then processed for WB analysis.

### FACS analysis

Bacteria were washed and then incubated for 1 h, at room temperature (RT), with polyclonal mouse antisera raised against the recombinant C2 fragment (working dilution 1:500). After several washing steps, samples were incubated for 30 min, at RT, with Alexa Flour 488-conjugated goat anti mouse IgG secondary antibody (working dilution 1: 1,000; Life Technologies). All washing steps and antibodies dilutions were performed using 1% (v/v) bovine serum albumin (Sigma) in PBS. Labeled bacteria were washed and fixed for 1 h at RT, using 2% (v/v) formaldehyde (Carlo Erba Reagents) in PBS. Samples were analyzed with BD FACS Canto II system (BD Bioscience).

### Proteomic analysis of polarized Calu-3 supernatant

For proteomic analysis, polarized Calu-3 supernatant was fractionated by anion exchange chromatography. Sample was dialyzed at 4 °C overnight against 50 mM Tris-HCl, pH = 8, using Snake Skin Dialysis Tubing, 3,500 MWCO, 22 mm (Thermo Scientific). Dialyzed sample was load on 1 mL HiTrap Q HP column (Ge Healthcare). Fractions were eluted increasing the ionic strength; a one-step NaCl gradient was performed using 50 mM Tris-HCl, 1 M NaCl, pH = 8. The AKTA FPLC system was used to control the NaCl gradient (from 0 M to 1 M NaCl). Protein fractions positive for proteolytic activity on NHBA protein or C2 fragment were pooled and precipitated in 10% (v/v) trichloroacetic acid and 0.04% (w/v) sodium dehoxicholate. Protein pellets were solubilized in 50 μl di 0.1% (w/v) RapigestTM (Waters, MA, USA) and 1mM DTT and 50 mM ammonium bicarbonate, boiled at 100°C for 10 min. After cooling down, 1μg of LysC/trypsin mix (Promega) was added and the reaction was performed overnight. Digestions were stopped with 0.1% final formic acid, desalted using OASIS HLB cartridges (Waters) as described by the manufacturer, dried in a Centrivap Concentrator (Labconco) and resuspended in 100 μl of 3% (v/v) acetonitrile (ACNcan) and 0.1% (v/v) formic acid. An Acquity HPLC instrument (Waters) was coupled on-line to a Q Exactive Plus (Thermo Fisher Scientific) with an electrospray ion source (Thermo Fisher Scientific). The peptide mixture (10 μl) was loaded onto a C18-reversed phase column Acquity UPLC peptide CSH C18 130Å, 1.7μm 1 x 150 mm and separated with a linear gradient of 28–85% buffer B (0.1% (v/v) formic acid in ACN) at a flow rate of 50 μL/min and 50 °C. MS data were acquired in positive mode using a data-dependent acquisition (DDA) dynamically choosing the five most abundant precursor ions from the survey scan (300–1600 *m/z*) at 70,000 resolution for HCD fragmentation. Automatic Gain Control (AGC) was set at 3E+6. For MS/MS acquisition, the isolation of precursors was performed with a 3 *m/z* window and MS/MS scans were acquired at a resolution of 17,500 at 200 *m/z* with normalized collision energy of 26 eV. The mass spectrometric raw data were analyzed with the PEAKS software ver. 8 (Bioinformatics Solutions Inc., ON, Canada) for de novo sequencing, database matching and identification. Peptide scoring for identification was based on a database search with an initial allowed mass deviation of the precursor ion of up to 15 ppm. The allowed fragment mass deviation was 0.05 Da. Protein identification from MS/MS spectra was performed against NCBInr *Homo sapiens* (Human) protein database (112,970,924 protein entries; 41,399,473,309 residues) combined with common contaminants (human keratins and autoproteolytic fragments of trypsin) with a FDR set at 0.1%. Enzyme specificity was set as C-terminal to Arg and Lys, with a maximum of four missed cleavages. N-terminal pyroGlu, Met oxidation and Gln/Asn deamidation were set as variable modifications.

### Intact mass measurement

In order to evaluate the cleavage site of epithelial cell proteases, the intact mass of His-tagged C-terminal portion of NHBA and C2 recombinant form were recovered with Ni^2+^ IMAC affinity enrichment after incubation with polarized Calu-3 cells. The acidified protein solutions were loaded onto a Protein Microtrap cartridge (from 60 to 100 pmols), desalted for 2 min with 0.1% (v/v) formic acid at a flow rate of 200 ml/min and eluted directly into the mass spectrometer using a step gradient of acetonitrile (55% (v/v) acetonitrile, 0.1% (v/v) formic acid). Spectra were acquired in positive mode on a SynaptG2 HDMS mass spectrometer (Waters) equipped with a Z-spray ESI source. The quadrupole profile was optimized to ensure the best transmission of all ions generated during the ionization process. Mass spectra were smoothed, centroided and deconvoluted using MassLynx vers. 4.1 (Waters).

## Results

### Proteases secreted by epithelial cells process NHBA-derived C2 fragment

Recombinant NHBA-derived C2 fragment exerts a toxic effect on endothelial cells altering endothelial permeability [**9**]. The recent finding that NHBA protein is more expressed and cleaved at lower temperatures than 37°C [**12**], which are temperatures encountered during *N*. *meningitidis* initial colonization of the upper respiratory tract, led us to hypothesize that C2 fragment could also alter the epithelial permeability and facilitate the traversal of *N*. *meningitidis* through the epithelium.

**Fig 1:**
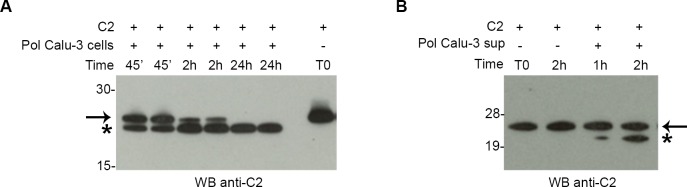
C2 fragment is processed by cell proteases secreted by polarized Calu-3 epithelial cells. (A) Western blot analysis of supernatants of polarized Calu-3 cells treated with 5 μM of recombinant C2 fragment. Analyzed samples derived from the permeability assay and they were collected at different time point (45’, 2h and 24h). (B) Western blot analysis of recombinant C2 fragment incubated with cell supernatants prepared from polarized Calu-3 cells. Samples were analyzed at different time point (1h and 2h). Polyclonal mouse sera against C2 fragment were used for blotting the membranes (A and B). The arrow indicates the C2 fragment and the asterisk indicates the shorter C-terminal fragment derived from the cleavage of epithelial cell proteases.

To test our hypothesis, we evaluated C2 fragment activity on polarized Calu-3 cells cultured on collagen-coated permeable membrane in cell culture inserts. Recombinant C2 fragment was added in the apical chamber and cellular integrity was assessed by measuring the permeability of epithelial cell monolayers to different fluorescent probes (BSA-FITC and Dextran-Texas Red) previously added in the apical chamber. No influence of the C2 fragment on permeability was observed comparing basal chamber fluorescence of treated monolayers with respect to untreated monolayers (S1 Fig). Consistent with this observation, organization of tight junction structures (TJs) was not altered by C2 fragment treatment, as revealed by the correct distribution of ZO-1 protein, a specific marker for TJs integrity (S1 Fig). Surprisingly, when we checked the stability of the C2 fragment during permeability assays by performing a Western blot analysis on treated cell supernatants, a second shorter band was observed (Fig 1A), indicating that epithelial cells were able to process the protein during the assays. This cleavage increased in a time-dependent manner. Of note, supernatants of epithelial cells alone were able to process the C2 fragment when incubated with the protein (Fig 1B), suggesting that cellular proteases responsible for the cleavage were secreted by epithelial cells.

### Epithelial cell proteases eliminate the C2 fragment Arg-rich region

**Fig 2:**
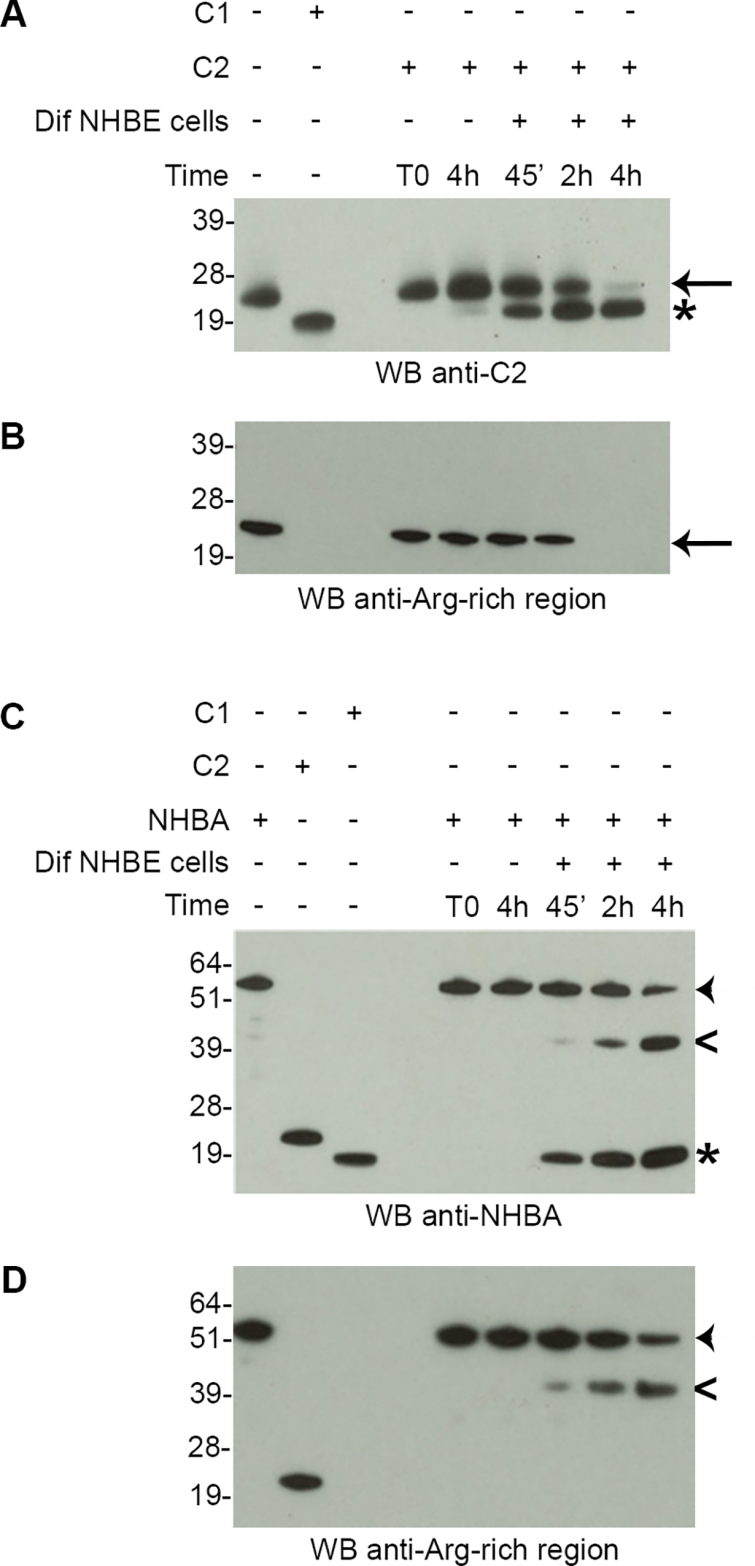
Proteases secreted by differentiated NHBE cells generate a NHBA C-terminal fragment devoid of the Arg-rich epitope. Western blot analysis of supernatants of differentiated NHBE cells treated with 5 μM of recombinant C2 fragment (A and B) or with 5 μM of recombinant NHBA full-length protein (C and D). Cell supernatants were collected at different time points (45’, 2h, and 4h). Polyclonal mouse sera against C2 fragment (A) or polyclonal mouse sera against NHBA full-length protein (C) or polyclonal rabbit sera against the Arg-rich region (B and D) were used for blotting the membranes. Recombinant C1 fragment, which corresponds to the C-terminal fragment generated upon cleavage of the NHBA protein by lactoferrin, was loaded as control for the blotting. The arrow indicates the recombinant C2 fragment. The arrowhead indicates the recombinant NHBA full-length protein. The asterisk and the open arrowhead indicates the C-terminal fragment and the N-terminal fragment, respectively, derived from the cleavage of epithelial cell proteases.

To validate our data on a more physiological system, we assessed the cleavage of both C2 fragment and NHBA protein using differentiated primary Normal Human Bronchial Epithelial (NHBE) cells. This primary cell line, grown on collagen-coated permeable membranes in cell culture inserts, develop a pseudo stratified epithelium composed of basal, ciliated and non-ciliated goblet cells [**16**]. In addition, it allowed us to exclude the possibility that the cleavage we detected was the result of contamination by serum proteases, since NHBE cells were cultivated and differentiated with serum-free medium. Similarly to what we observed with polarized Calu-3 cells, both the C2 fragment and the NHBA protein were cleaved when incubated with differentiated NHBE cells (Fig 2) or with NHBE apical washes (S2 Fig). For both proteins, the cleavage increased overtime and resulted in the generation of a C-terminal fragment that is devoid of the Arg-rich region epitope (Fig 2B and 2D), thus eliminating the domain essential for C2-mediated toxic effect.

### NHBA cleavage by epithelial cell proteases occurs on live bacteria

**Fig 3:**
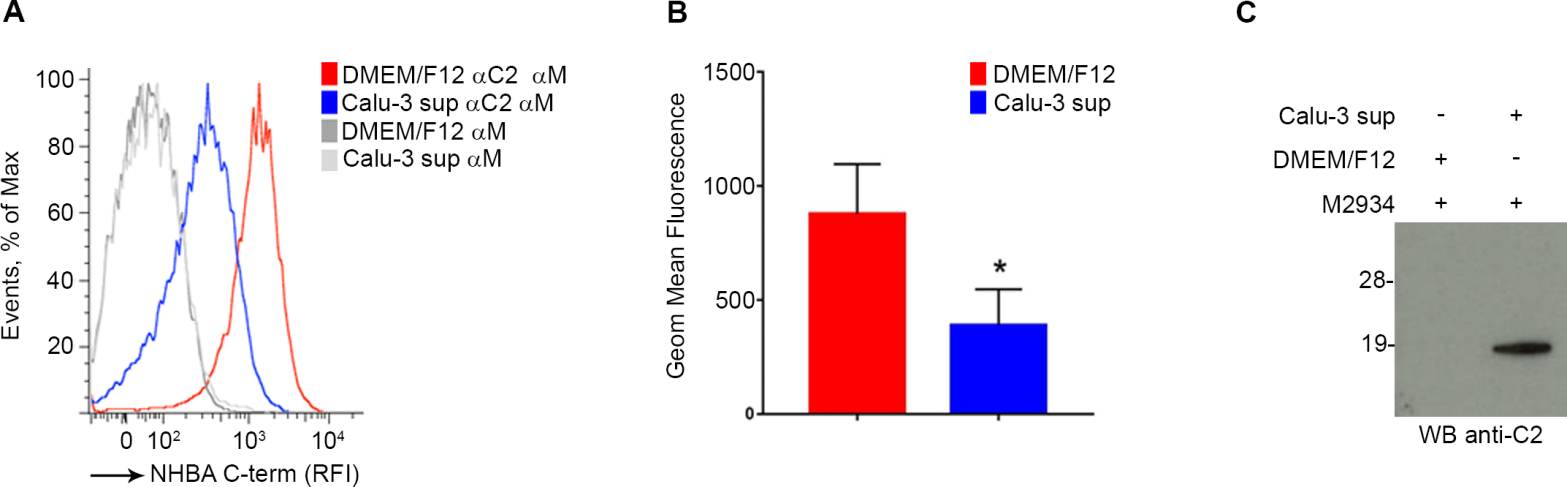
NHBA protein is cleaved by epithelial cell proteases on live bacteria. (A) and (B) Flow cytometric analysis (FACS) of M2934 strain stained with anti NHBA C-terminal antibody (α-C2) followed by anti–mouse Ig FITC-labeled secondary antibodies (α-M). Bacteria were incubated for two hours with epithelial cell supernatant (Calu-3 sup) or medium (DMEM/F12) and the presence of C-terminal domain on surface-exposed NHBA proteins in M2934 strain was evaluated. A representative FACS profile obtained by plotting histograms of relative fluorescence intensity (RFI) is shown (Panel A). The data are expressed as geometric mean of the fluorescence intensity (Panel B) and show the means of results from three independent experiments. Error bars denote standard deviation. Statistical analysis was performed using an unpaired, two-tailed T-test (p<0.03); C) Western blot analysis of concentrated supernatants derived from M2934 bacteria incubated for 2 hours with epithelial cell supernatant (Calu-3 sup) or medium (DMEM/F12). Polyclonal mouse sera against C2 fragment were used for blotting the membrane.

To verify our *in vitro* observations with recombinant proteins also on native protein expressed on live bacteria, we used natural strain M2934 expressing NHBA peptide 5, which is not cleaved by meningococcal proteases (data not shown). Thus, this strain allowed us to focus exclusively on NHBA cleavage by epithelial cell proteases. Bacteria were incubated either with Calu-3 cell supernatant or with cell medium, as negative control. After 2h, samples were stained with an antibody directed to the C-terminus of NHBA conjugated to FITC, and the percentage of bacterial cells exposing the C-terminal domain of NHBA on their surface was measured by flow cytometry analysis. As shown in Fig 3A, we observed a negative shift in fluorescence intensity when we compared bacteria incubated with epithelial cell supernatant to bacteria incubated with medium, indicating that treatment with cell supernatant led to a decrease of C-terminal domain on surface-exposed NHBA proteins in M2934 strain. This reduction was statistically significant (Fig 3B). The evidence that epithelial cell supernatant cleaved NHBA protein on live bacteria was further demonstrated by Western blot analysis. Supernatants derived from bacteria incubated for 2 hours with epithelial cell supernatant or medium were collected, concentrated and tested for the presence of NHBA C-terminal fragment. As shown in Fig 3C, NHBA C-terminal fragment was only detected in the concentrated supernatant of bacteria treated with epithelial cell supernatant, corroborating that the removal of NHBA C-terminal domain from bacterial surface by epithelial cell proteases resulted in the accumulation of C-terminal fragment into the supernatant.

### Cleavage site of epithelial cell proteases is located immediately downstream the Arg-rich region

**Fig 4:**
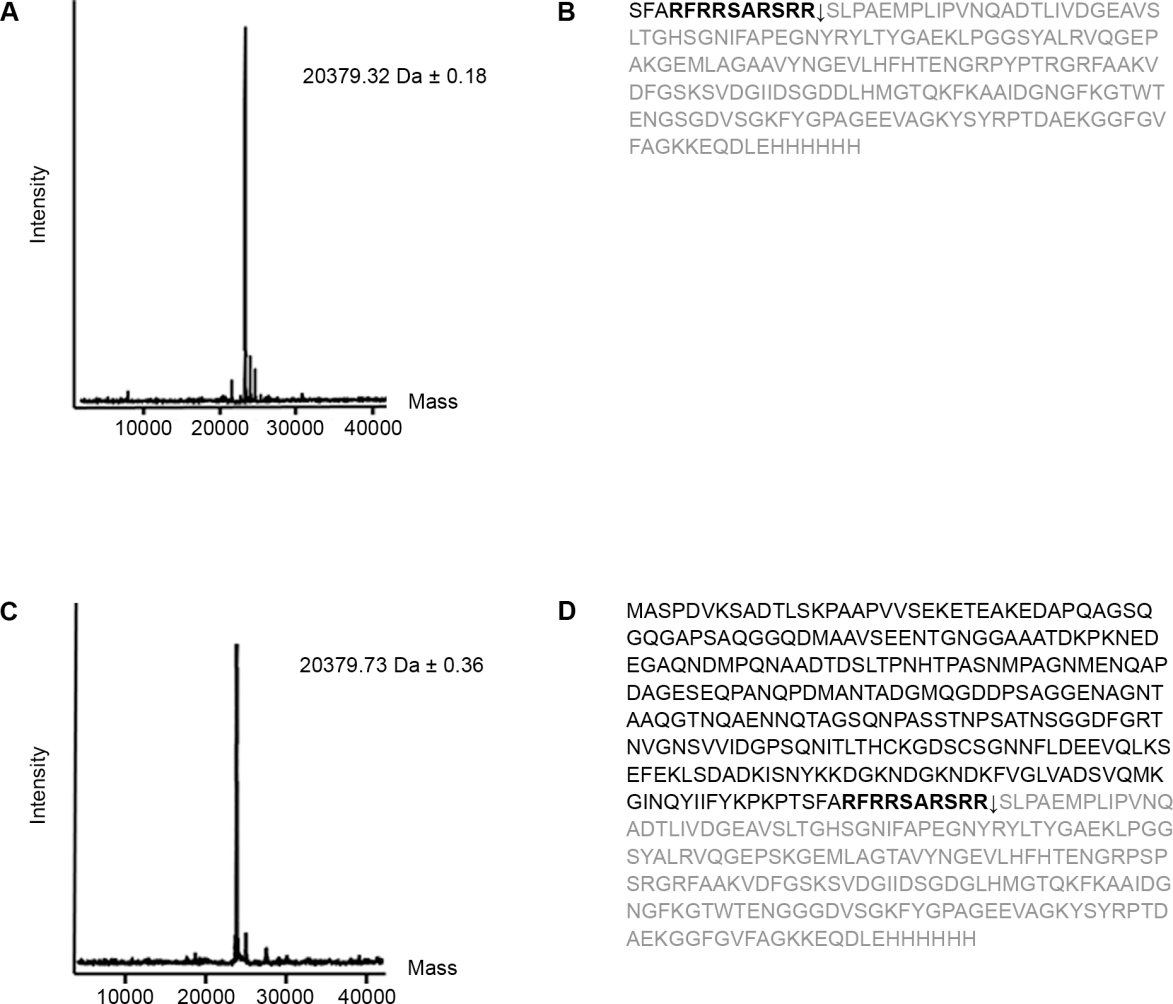
Epithelial cell proteases cleave NHBA and C2 fragment immediately downstream the Arg-rich region. (A) and (C) Intact mass measurement of C-terminal fragments generated by the cleavage of epithelial cells using recombinant C2 fragment (A) or NHBA full-length protein (C) as substrates. Experimental mass values measured after deconvolution are indicated. B and D) Amino acid sequence of recombinant C2 fragment (B) and NHBA full-length protein (D). Arg-rich region is indicated in bold. Cleavage site of epithelial cell proteases is indicated with a black arrow. Amino acid sequence of newly generated C-terminal fragment is highlighted in gray.

In a first effort to define the site within the C2 fragment or the NHBA protein where epithelial cell proteases cleave, we examined if the presence of the Arg-rich region was necessary for the cleavage. To this end, we used a recombinant NHBA mutant protein [**7**], wherein all arginines of the Arg-rich region were substituted with glycines (mRR). After incubating the protein with polarized Calu-3 cells, the cleavage was assessed at different time points by Western blot analysis. At all-time points tested, the mRR mutant protein was not cleaved by polarized Calu-3 cell proteases (S3 Fig), indicating that the presence of Arg-rich region was necessary for the processing of NHBA by epithelial cell proteases. This result suggested also that the cleavage by epithelial cell proteases occurred somewhere in the arginine-rich region. To further define the cleavage site of epithelial cell proteases, the intact mass of C-terminal fragments generated by either the cleavage of C2 fragment or the cleavage of NHBA protein was measured by mass spectrometry. In both case, a mass of 20379 Da was detected corresponding to a C-terminal fragment that started at Ser14 or Ser281, respectively (Fig 4). Thus, we concluded that the cleavage site of epithelial cells was located immediately downstream the Arg-rich region, and corresponds to the cleavage site of human lactoferrin [**7**].

### EDTA abolishes NHBA cleavage by epithelial cell proteases

To identify the epithelial cell protease responsible for NHBA cleavage, we performed an ion exchange chromatography of polarized Calu-3 cell supernatant in order to enrich a fraction of cell supernatant with the protease of our interest. Fractions were eluted increasing the ionic strength and then examined for their ability to cleave both recombinant C2 fragment and NHBA full-length protein. Therefore, recombinant proteins were incubated overnight with each eluted fraction and the cleavage was assessed by SDS-PAGE analysis. Cleavage was observed with 4 fractions (S4 Fig); however, only 2 fractions (17 and 18) were selected for further analysis since they showed greater cleavage efficiency and likely contained more protease molecules of our interest. These fractions were pooled, subjected to trypsin digestion and analyzed by mass spectrometry. From the list of proteins identified by mass spectrometry (S1 Table), we selected proteins annotated as proteases and secreted by cells. Six protease candidates were identified and they are listed in Table 1.

**Table 1:** List of epithelial protease candidates responsible for NHBA cleavage.

Protease	Activity	Co-factor	Localization	Specific inhibitor
**ADAM9**	Metalloendopeptidase	Zn^2+^	Isoform 2 secreted	GI254023X
**CATHEPSIN D**	aspartic endopeptidase cysteine endopeptidase serine endopeptidase		Secreted and Lysosome	Pepstatin A
**CATHEPSIN L1**	cysteine endopeptidase serine endopeptidase		Secreted	E-64
**COMPLEMENT FACTOR B**	serine endopeptidase	Mg^2+^ (§)	Secreted	
**LACTOFERRIN**	serine endopeptidase		Secreted	
**PROSTASIN**	serine endopeptidase		Light chain secreted	

(§) In presence of Mg^2+^, complement factor B binds to hydrolyzed form of complement C3 (C3 (H_2_O)) or C3b. This complex is recognized by the complement factor D or kallikrein that cleaves and activates factor B, generating the C3-convertase of the alternative complement pathway.

**Fig 5:**
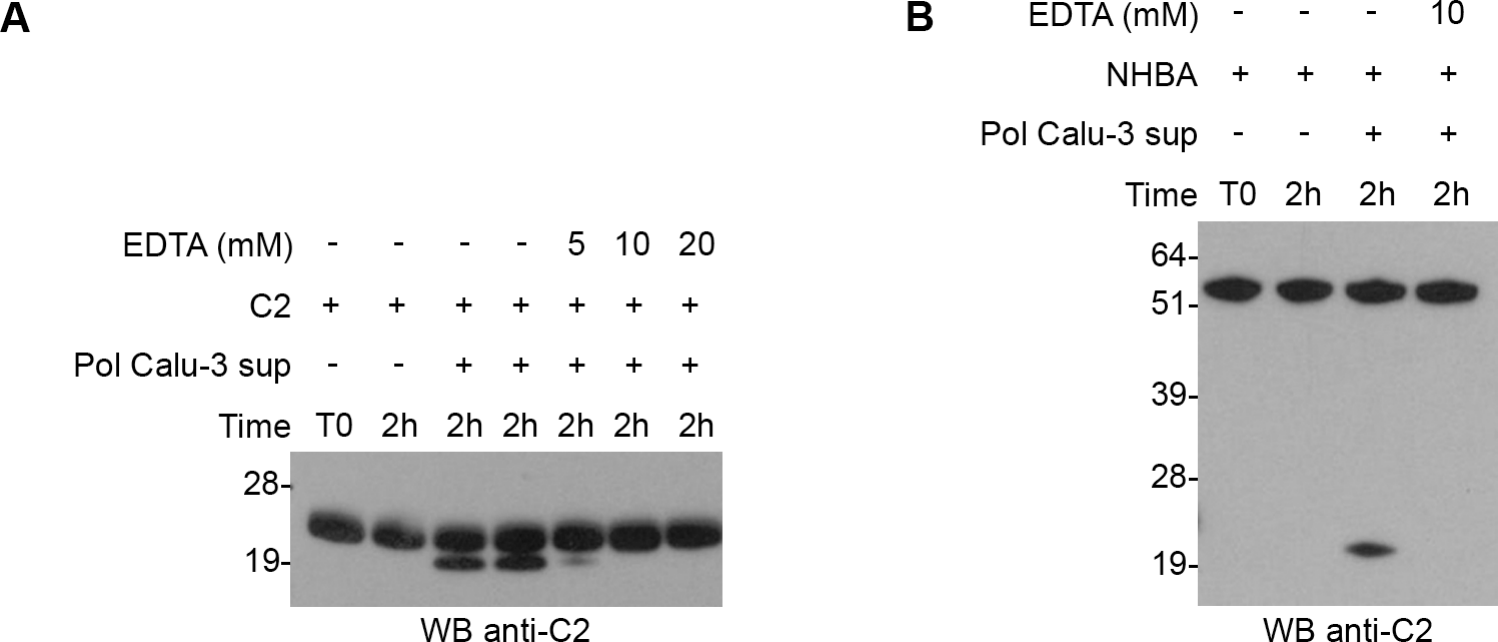
EDTA completely inhibits NHBA cleavage by epithelial cell proteases. Western blot analysis of recombinant C2 fragment (A) or NHBA full-length protein (B) incubated for 2h with polarized Calu-3 cell supernatants that were pre-treated or not with EDTA for 30 min. Polyclonal mouse sera against C2 fragment were used for blotting the membranes.

Specific protease inhibitors were used to identify the protease responsible for the cleavage of C2 fragment and NHBA full-length protein. The following specific inhibitors were chosen and tested at several concentrations: GI254023X [**17**], Pepstatin A and E-64 (S5 Fig). With this approach, we ruled out the possibility that ADAM9, Cathepsin D and L1 could be the proteases responsible for the cleavage, since the cleavage was still occurring. EDTA, a chelate agent of divalent and trivalent positive ions (*i*.*e*. Ca^2+^, Mg^2+^, Zn^2+^, Fe^3+^), was the only inhibitor able to block the activity of the epithelial cell protease of our interest (Fig 5). A concentration range of 1-10mM of EDTA was tested, and at 5mM the cleavage of epithelial cell supernatant was completely inhibited.

From the list of candidates, the protease activity dependent on positive ions, and that could be affected by EDTA treatment, was the human complement factor B. Complement factor B in the presence of Mg^2+^ interacts with the hydrolyzed form of complement C3 (C3(H2O)) or C3b. This complex is then recognized by complement factor D or kallikrein that cleaves and activates factor B, and thus generates the C3-convertase of the alternative complement pathway [**18**; **19**]. Human complement component C3 was also identified by mass spectrometry in the selected fractions of polarized Calu-3 cell supernatant (S1 Table). The expression of both complement component C3 and factor B by epithelial cells was also verified at the mRNA level (S6 Fig).

### Alternative pathway C3-convertase processes NHBA protein

**Fig 6:**
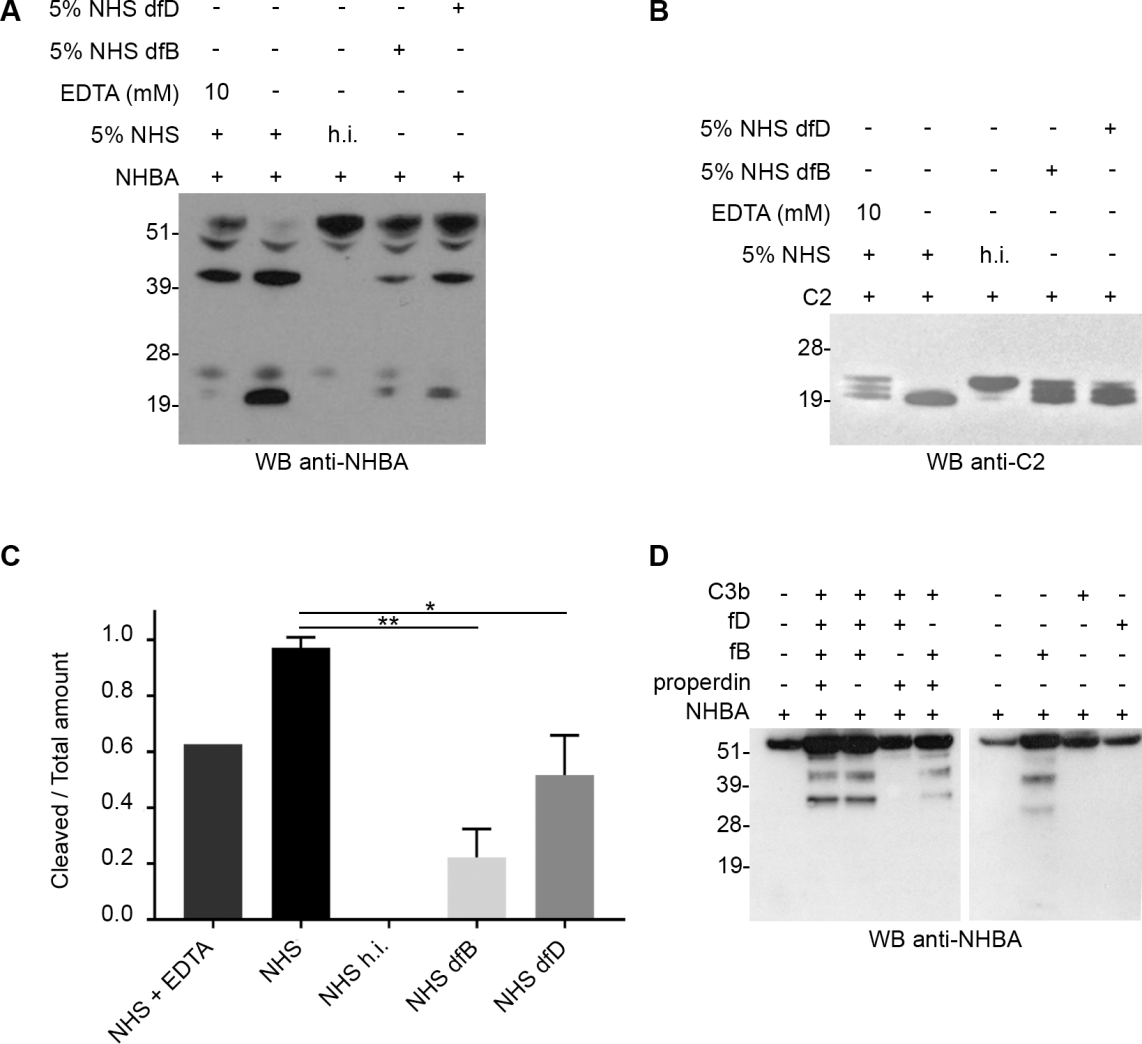
C3-convertase of the alternative complement pathways: A human protease responsible for NHBA cleavage. Western blot analysis of recombinant C2 fragment (A) or NHBA full-length protein (B) incubated for 1 hour with Normal Human Serum (NHS), NHS pre-treated with 10mM EDTA, NHS depleted of factor B (NHS dfB), NHS depleted of factor D (NHS dfD) and NHS heat inactivated (h.i.), as negative control. Polyclonal mouse sera against C2 fragment (A) or NHBA full-length protein (B) were used for blotting the membranes; C) Quantification of NHBA cleavage by densitometric analysis of western blot bands. Relative levels of NHBA cleavage were estimated by quantifying the ratio between N-terminal fragment/total amount of NHBA. Data show the means of results from three independent experiments. Error bars denote standard deviation. Statistical analysis was performed using an unpaired, two-tailed T-test (p<0.01; p<0.04); D) Western blot analysis of NHBA full-length protein incubated for 1h with purified complement components: C3b, factor B (fB) and factor D (fD). Properdin was included in the assay to stabilize the C3bBb complex [**20**]. Polyclonal mouse sera against NHBA full-length protein were used for blotting the membranes.

To further confirm that C3-convertase of the alternative complement pathway was a protease responsible for the processing of C2 fragment and NHBA full-length protein, we decided to use Normal Human Serum (NHS) since in NHS complement components are highly concentrated and it is where they play their main physiological role. The cleavage was tested in three different conditions for which the C3-convertase of the alternative complement pathway could not be formed: (i) NHS was treated with EDTA in order to sequestrate Mg^2+^ and prevent the interaction between factor B and C3(H2O); (ii) NHS was depleted of factor B; (iii) NHS was depleted of factor D in order to prevent the activation of the C3(H2O)B complex. Regarding to NHS, in all the three conditions tested, in which the formation of C3-convertase was inhibited, we observed a reduction of the cleavage of both substrate proteins and the generations of two distinct smaller C-terminal fragments (Fig 6), compatible with the kallikrein cleavage pattern (S7 Fig). This reduction was statistically different (Fig 6C). Moreover, the processing of NHBA protein was observed after the incubation with purified complement components, previously mixed to generate *in vitro* the C3-convertase of the alternative complement pathway (Fig 6D). The sole presence of factor B seemed to be sufficient for the cleavage even though the reaction efficiency was low (Fig 6D, lane 7). Taking together, these results indicated that C3-convertase of the alternative complement pathway is another human protease responsible for the cleavage of C2 fragment and NHBA protein.

## Discussion

**Fig 7:**
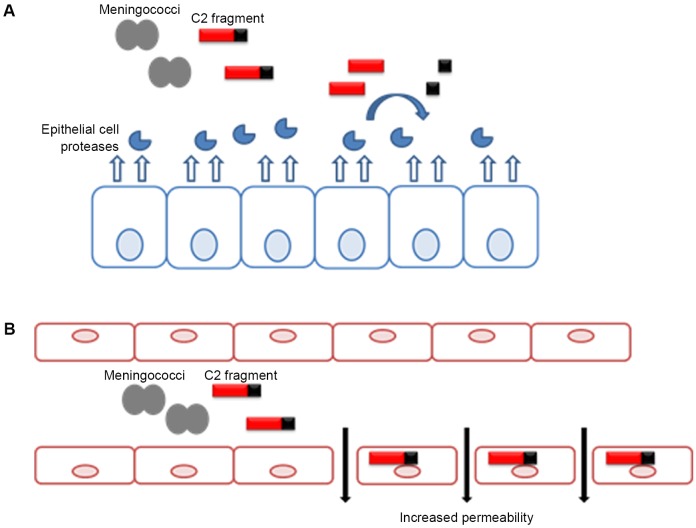
Differential outcomes during C2 fragment interaction with host cells depending on cell type. (A) Epithelial cells constantly produce proteases that are able to process the C2 fragment released by meningococci. This cleavage prevents C2 fragment-induced toxic effect by eliminating the Arg-rich domain, and thus the integrity of the epithelium is maintained. (B) Under *in vitro* condition, endothelial cells do not express proteases that process the C2 fragment. Thus, C2 fragment produced by meningococci interacts with endothelial cells and induces the alteration of endothelial integrity.

In the present study, using an *in vitro* model of human airway epithelium, we demonstrated that C2 fragment did not alter the integrity of epithelial monolayers, and this was in agreement with previous findings showing that *N*. *meningitidis* traverses the epithelial barrier without disrupting the junctional structures [**3**; **4**; **5**]. Unexpectedly, we discovered that epithelial cells were able to process the C2 fragment converting it into a shorter fragment. Proteolysis of C2 fragment by epithelial cells eliminated the Arg-rich region, the putative docking domain responsible for the interaction of C2 fragment with host cells [**9**], likely preventing its biological activity. Therefore, this cleavage could represent a host defense mechanism against C2 fragment-induced toxic effect. Based on these findings we propose a model, depicted in Fig 7, to explain different outcomes as a result of the interaction between C2 fragment and host cells depending on the cell type. In a subset of hyper virulent strains, NHBA protein is cleaved by meningococcal proteases and the C2 fragment, a potential virulence factor, is released from bacterial surface. Acting as a first line of defense against meningococcal invasion, the airway epithelium constantly produces proteases that inactivate the C2 fragment activity. In contrast, under the *in vitro* conditions tested endothelial cells do not express proteases that are promptly able to process the C2 fragment, allowing the C2 fragment to exert its toxic effect that results in the perturbation of endothelial integrity.

Investigating further the cleavage by epithelial cells using different models of study, we found that epithelial cells from the upper respiratory tract constantly secrete proteases responsible for a rapid cleavage of both C2 fragment and NHBA full-length protein. We also demonstrated that processing of NHBA protein occurred on live bacteria. Removal of NHBA C-terminal domain from bacterial surface resulted in the generation of an N-terminal fragment containing the Arg-rich region disclosed as new C-terminal domain, which might remain exposed on bacterial surface.

Finally we demonstrated that, in addition to kallikrein and lactoferrin [**7**; **11**], C3-convertase of the alternative complement pathway is a human protease able to process both C2 fragment and NHBA full-length protein. Formation of alternative pathway C3-convertase on the surface of *N*. *meningitidis* certainly occurred in human blood, and it might also occur during meningococcal colonization of the nasopharynx. In agreement with previous studies on expression profiling of human epithelial cells of the upper respiratory tract [**21**; **22**; **23, 24**], we reported, indeed, that Calu-3 epithelial cells expressed and secreted complement component C3 and Factor B. As a result of tick-over mechanism of C3 molecule [**19**], C3(H_2_O) can interact with factor B in presence of Mg^2+^ and forms the C3-convertase of the alternative complement pathway. This complex needs to be activated by the cleavage of Factor D or kallikrein [**18**; **25**]. In this study, neither Factor D nor kallikrein has been detected in the fractions of epithelial cell supernatants responsible for NHBA cleavage by mass spectrometry analysis. However, it is well known that tissue kallikrein 1 is expressed by tracheobronchial submucosal glands in human airways [**26**] and its production increases under inflammatory conditions, such as asthmas and viral infection [**27**; **28**; **29**]. Thus, C3-convertase of alternative pathway could be activated on mucosal surface at least under these conditions. With *N*. *meningitidis*, a concomitant inflammation of the airway epithelium, might lead to the formation of alternative pathway C3-convertase on bacterial surface. Once activated, it might cleave surface-exposed NHBA protein.

Removal of NHBA C-terminal domain from bacterial surface by C3-convertase of the alternative complement pathway might affect the complement activation acting on two different steps of the complement cascade: (i) the antibody-mediated activation of the classical pathway and (ii) the amplification loop of the alternative pathway. First of all, if the C-terminal domain of NHBA protein is removed from the bacterial surface, antibodies raised against the C-terminal domain will not be able to initiate the complement cascade. This mechanism could be extended to all proteases that process surface-exposed NHBA protein, meningococcal and host proteases. In spite of this, antibodies directed to the N-terminal domain will be functional also after the processing of NHBA protein, since N-terminal fragment remains anchored to the bacterial surface. Using rabbit complement as source of complement, it has been shown that NHBA cleavage, by either meningococcal NalP protease or by human lactoferrin, does not significantly affect the NHBA-mediated bactericidal activity, since bactericidal titers varied within one dilution [**7**]. However, further confirmations using human complement as source of complement need to be carried out, in order to exclude the possibility that removal of NHBA C-terminal domain is a human species-specific mechanism to avoid complement killing, as it has been discovered to be for fHbp and NspA proteins [**30**; **31**]. Secondly, involvement of alternative pathway C3-convertase in the cleavage of NHBA protein from bacterial surface might interfere with the amplification loop of the alternative pathway. The amplification loop is the balance between two separate competing cycles: C3b–C3 convertase formation and C3b breakdown, and it contributes to the overall complement response pushing the complement cascade to the formation of C5-convertase that finally will lead to the lyses of bacteria trough the formation of MAC complex on bacterial surface [**19**]. Since the alternative complement C3-convertase has a very short half-life (90 seconds), if it is occupied with the processing of NHBA protein on bacterial surface instead of activate more C3 to C3b, the balance will likely shift in favor of the C3b breakdown cycle dampen the complement cascade. This might represent an additional mechanism used by *N*. *meningitidis* to evade the complement system and survive in human blood.

Overall, these conclusive speculations raise the question whether the direct interaction between NHBA protein and alternative pathway C3-convertase may limit the functionality of Serum Bactericidal Antibody (SBA) assay, which is used for measuring the concentration of functional antibodies induced by NHBA antigen, leading to an underestimation of the real functionality of these antibodies. Since antibodies against NHBA have been shown to inhibit adhesion of *N*. *meningitidis* to epithelial cells [**10]**, inhibition of meningococcal adhesion to epithelial cells by anti-NHBA sera can be taken in consideration as an additional assay for measuring their functionality. A recent report, showing that vaccination with Bexsero reduced meningococcal carriage rates of diverse circulating strains during 12 months after vaccination [**32]**, highlights the fact that the Bexsero vaccine likely confers protection against meningococcal disease acting at different stages of the infection, including colonization. Antibodies induced by NHBA antigen may contribute to protection mainly at this initial stage of the infection by inhibiting colonization of NHBA-expressing bacteria [**10]**. Therefore, it will be important to evaluate the contribution of NHBA antigen in vaccine efficacy at the level of meningococcal colonization, and not only for its ability to induce bactericidal antibodies.

## Supporting information

S1 Fig C2 Fragment does not impair the integrity of the epithelium. A) and B) Recombinant C2 fragment does not alter the permeability of the epithelium. In the permeability assay, polarized Calu-3 cells were cultured on collagen-coated permeable membrane in cell culture inserts. Recombinant C2 fragment was added in the apical chamber and cellular integrity was assessed by measuring the passage of a fluorescent probe, previously added in the apical chamber, into the lower chamber, at various time intervals. Two different probes with different molecular weights were used: BSA-FITC (A) and 10,000 MW Dextran-Texas Red (B). TcdA, *Clostridium difficile* toxin, was included in the assay as positive control. The permeability of cell monolayers for a probe was quantified calculating the ∆ mean of fluorescent intensity normalized versus pre-treatment values. C) Recombinant C2 fragment does not alter the organization of tight junction structures (TJs). Immunofluorescence analysis of ZO-1 protein distribution, a specific marker for TJs, (red staining) in C2 fragment treated (upper panel) or untreated (lower panel) polarized Calu-3 cells was performed. Cell nuclei were detected with DAPI (blue staining). No significant changes in ZO-1 distribution were observed indicating that TJs integrity was preserved.
(TIF)

S2 Fig C2 fragment and NHBA protein are processed by cell proteases secreted by differentiated NHBE epithelial cells. Western blot analysis of recombinant C2 fragment or NHBA full-length protein incubated with cell supernatants prepared from differentiated NHBE cells. Samples were analyzed at different time points (45’, 1h, 2h and 4h). Polyclonal mouse sera against C2 fragment (A) or polyclonal mouse sera against NHBA full-length protein (B) were used for blotting the membranes. The arrow indicates the recombinant C2 fragment. The arrowhead indicates the recombinant NHBA full-length protein. The asterisk and the open arrowhead indicates the C-terminal fragment and the N-terminal fragment, respectively, derived from the cleavage of epithelial cell proteases.
(TIF)

S3 Fig mRR NHBA mutant protein is not cleaved by epithelial cell proteases. Western blot analysis of supernatants of polarized Calu-3 cells treated with 5 μM of recombinant mRR NHBA mutant protein. Samples were collected at different time points (45’, 2h, 4h and 24h). Polyclonal mouse sera against NHBA full-length protein were used for blotting the membrane. Recombinant NHBA C-terminal fragments, C2 and C1, were loaded as controls for the blotting.
(TIF)

S4 Fig Identification of cell supernatant fractions enriched with the epithelial cell protease responsible for NHBA cleavage. Ion exchange chromatography of polarized Calu-3 cell supernatant A) Chromatogram shows elution of fractions (in red), protein absorbance at 280nm (in blue), salt concentration (in green) and conductivity (in brown). B and C) SDS-PAGE analysis of each eluted fraction incubated overnight (o/n) with 5 μM of recombinant C2-fragment (B) or with 5 μM of recombinant NHBA full length protein (C). Proteins were stained with blue coomassie.
(TIF)

S5 Fig Screening of protease inhibitors. Western blot analysis of recombinant C2 fragment (A) or NHBA full-length protein (B) incubated for 2 hours with Calu-3 cell supernatants that were pre-treated or not with protease inhibitors for 30 minutes. Protease inhibitors tested: EDTA, Leupeptin (Leu), Pepstatine A (Pep), GI254023X and E-64. Polyclonal mouse sera against C2 fragment (A) or against NHBA full-length protein (B) were used for blotting the membranes.
(TIF)

S6 Fig Calu-3 epithelial cells express complement component C3 and factor B. Agarose gel electrophoresis analysis of expression of human CFB and C3 genes in Calu-3 cells (A) and polarized Calu-3 cells (B). GAPDH was used as internal positive control. Total RNA was isolated from epithelial cells, retro transcribed with oligo(dT) and cDNA were used as templates for PCR amplification. For each gene analyzed, specific oligonucleotides amplified part of the mRNA (GAPDH: 518 nt; CFB: 885 nt; C3: 408 nt).
(TIF)

S7 Fig EDTA does not inhibit the activity of kallikrein. A) SDS-PAGE analysis of recombinant C2 fragment incubated overnight with plasma-purified kallikrein pre-treated or not with EDTA. Proteins were stained with blue coomassie. B) Western blot analysis of recombinant NHBA full-length protein incubated overnight with plasma-purified kallikrein pre-treated or not with EDTA. Polyclonal mouse sera against NHBA full-length protein were used for blotting the membrane.
(TIF)

S1 Table List of proteins contained in the selected fractions of polarized Calu-3 cell supernatant identified by mass spectrometry. (PDF)

S1 Text Supporting materials and methods. (DOCX)
