# Supplementary figures and images for: Neisseria Heparin Binding Antigen is targeted by the human alternative pathway C3-convertase
Source: PLoS One. 2018 Mar 26;13(3):e0194662. doi: 10.1371/journal.pone.0194662 (PMC5868813; doi:10.1371/journal.pone.0194662)

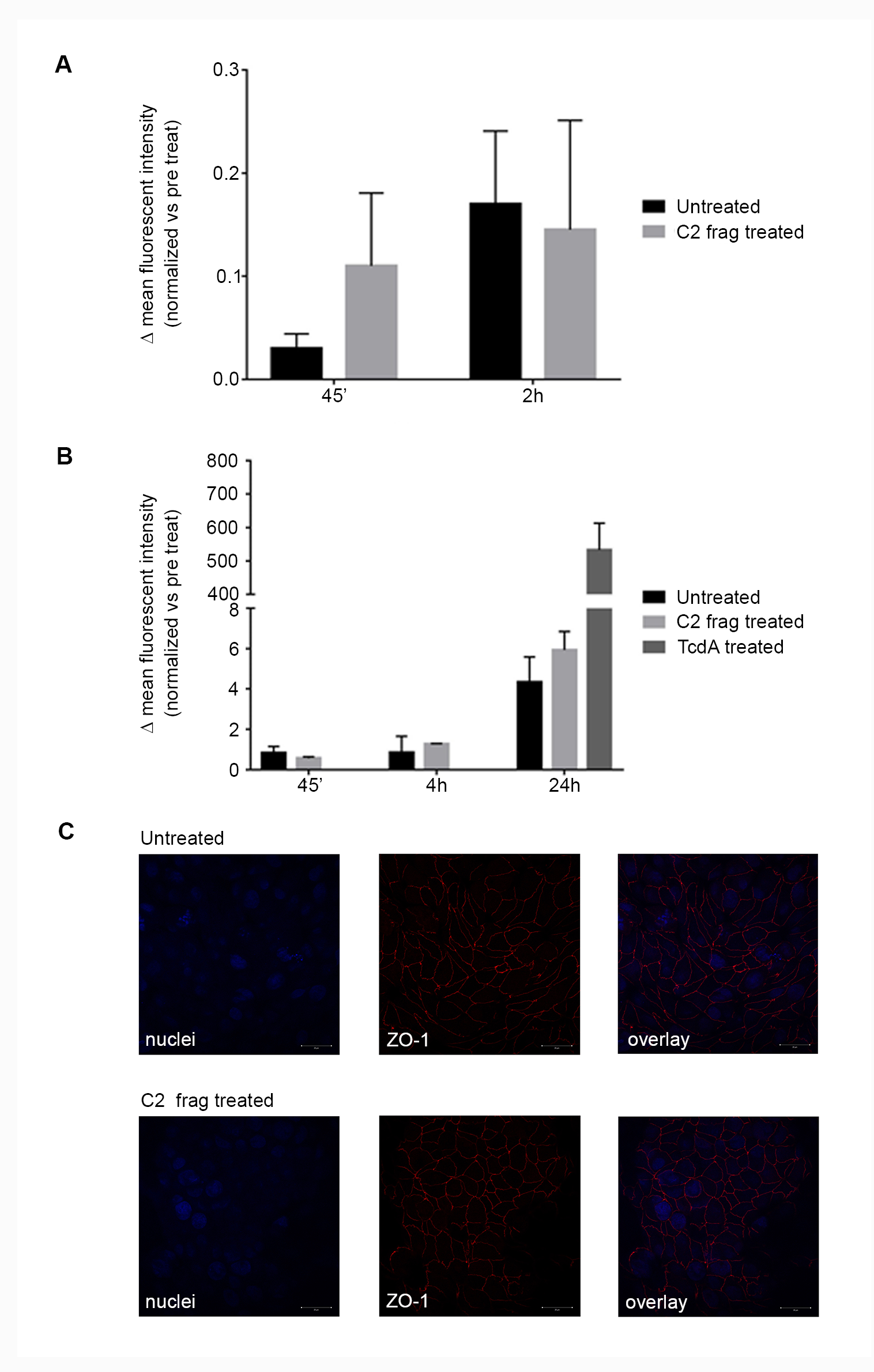

Supplement: S1 Fig — A) and B) Recombinant C2 fragment does not alter the permeability of the epithelium. In the permeability assay, polarized Calu-3 cells were cultured on collagen-coated permeable membrane in cell culture inserts. Recombinant C2 fragment was added in the apical chamber and cellular integrity was assessed by measuring the passage of a fluorescent probe, previously added in the apical chamber, into the lower chamber, at various time intervals. Two different probes with different molecular weights were used: BSA-FITC (A) and 10,000 MW Dextran-Texas Red (B). TcdA, Clostridium difficile toxin, was included in the assay as positive control. The permeability of cell monolayers for a probe was quantified calculating the ∆ mean of fluorescent intensity normalized versus pre-treatment values. C) Recombinant C2 fragment does not alter the organization of tight junction structures (TJs). Immunofluorescence analysis of ZO-1 protein distribution, a specific marker for TJs, (red staining) in C2 fragment treated (upper panel) or untreated (lower panel) polarized Calu-3 cells was performed. Cell nuclei were detected with DAPI (blue staining). No significant changes in ZO-1 distribution were observed indicating that TJs integrity was preserved. (TIF) [file pone.0194662.s001.tif]

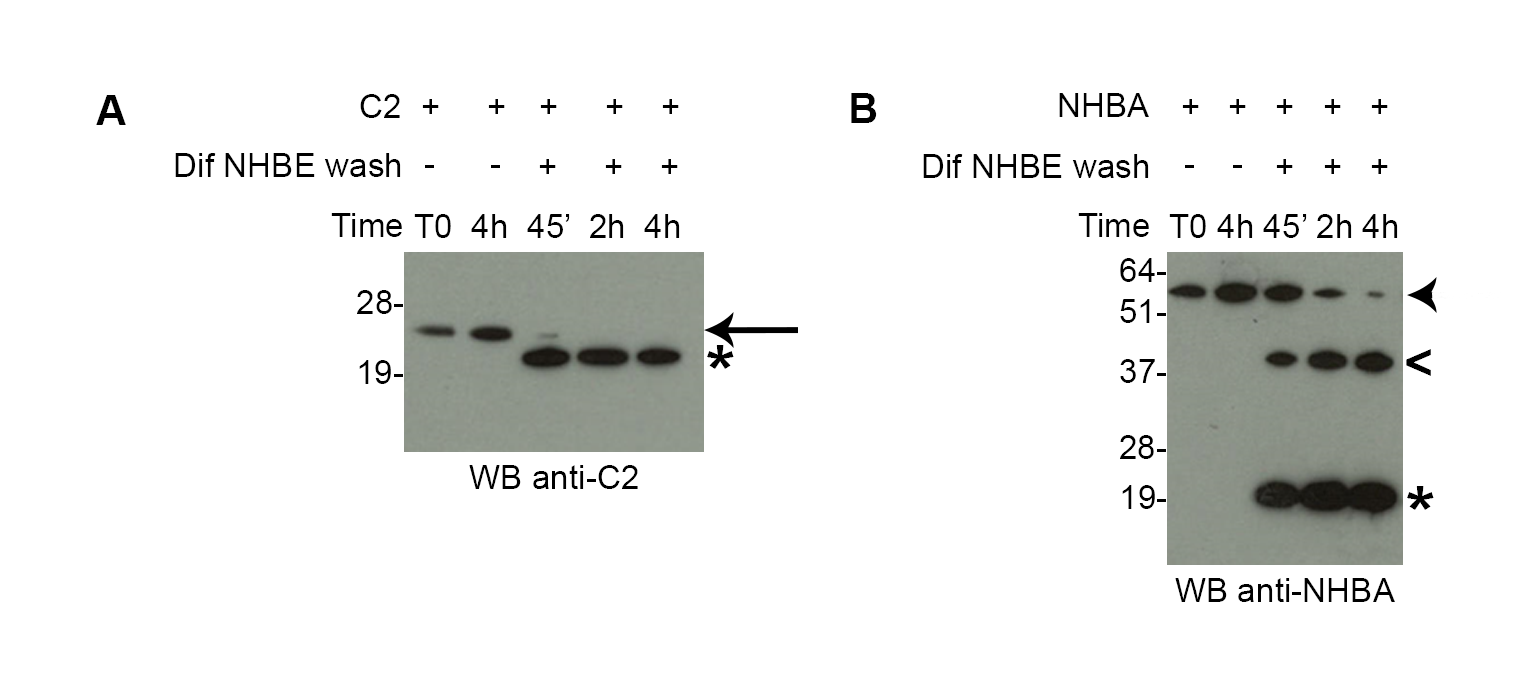

Supplement: S2 Fig — Western blot analysis of recombinant C2 fragment or NHBA full-length protein incubated with cell supernatants prepared from differentiated NHBE cells. Samples were analyzed at different time points (45’, 1h, 2h and 4h). Polyclonal mouse sera against C2 fragment (A) or polyclonal mouse sera against NHBA full-length protein (B) were used for blotting the membranes. The arrow indicates the recombinant C2 fragment. The arrowhead indicates the recombinant NHBA full-length protein. The asterisk and the open arrowhead indicates the C-terminal fragment and the N-terminal fragment, respectively, derived from the cleavage of epithelial cell proteases. (TIF) [file pone.0194662.s002.tif]

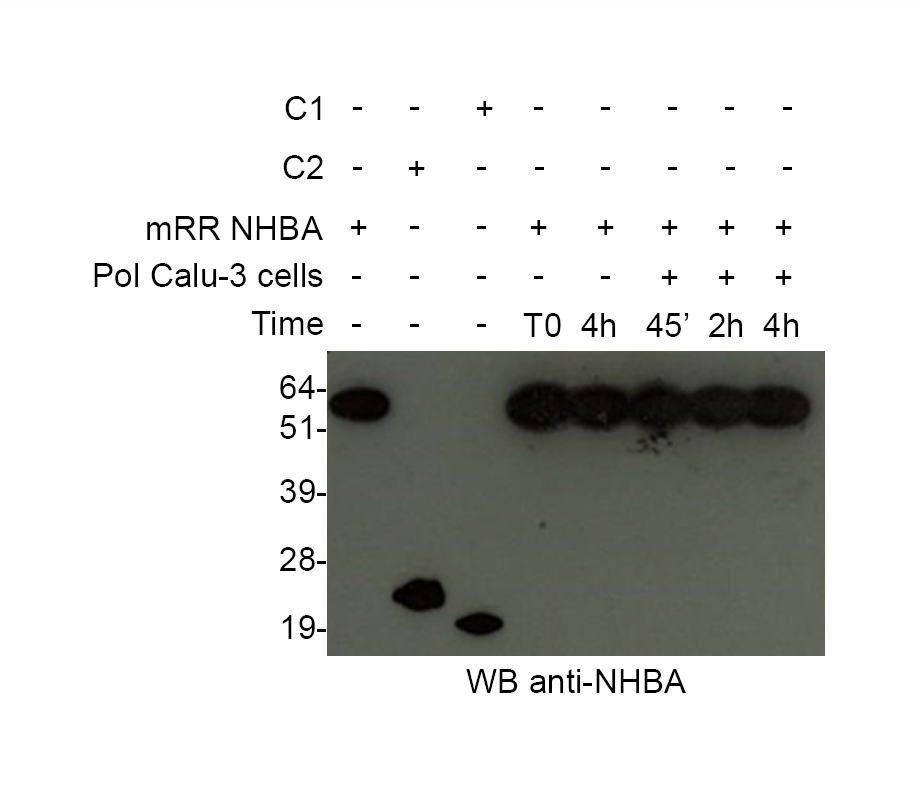

Supplement: S3 Fig — Western blot analysis of supernatants of polarized Calu-3 cells treated with 5 μM of recombinant mRR NHBA mutant protein. Samples were collected at different time points (45’, 2h, 4h and 24h). Polyclonal mouse sera against NHBA full-length protein were used for blotting the membrane. Recombinant NHBA C-terminal fragments, C2 and C1, were loaded as controls for the blotting. (TIF) [file pone.0194662.s003.tif]

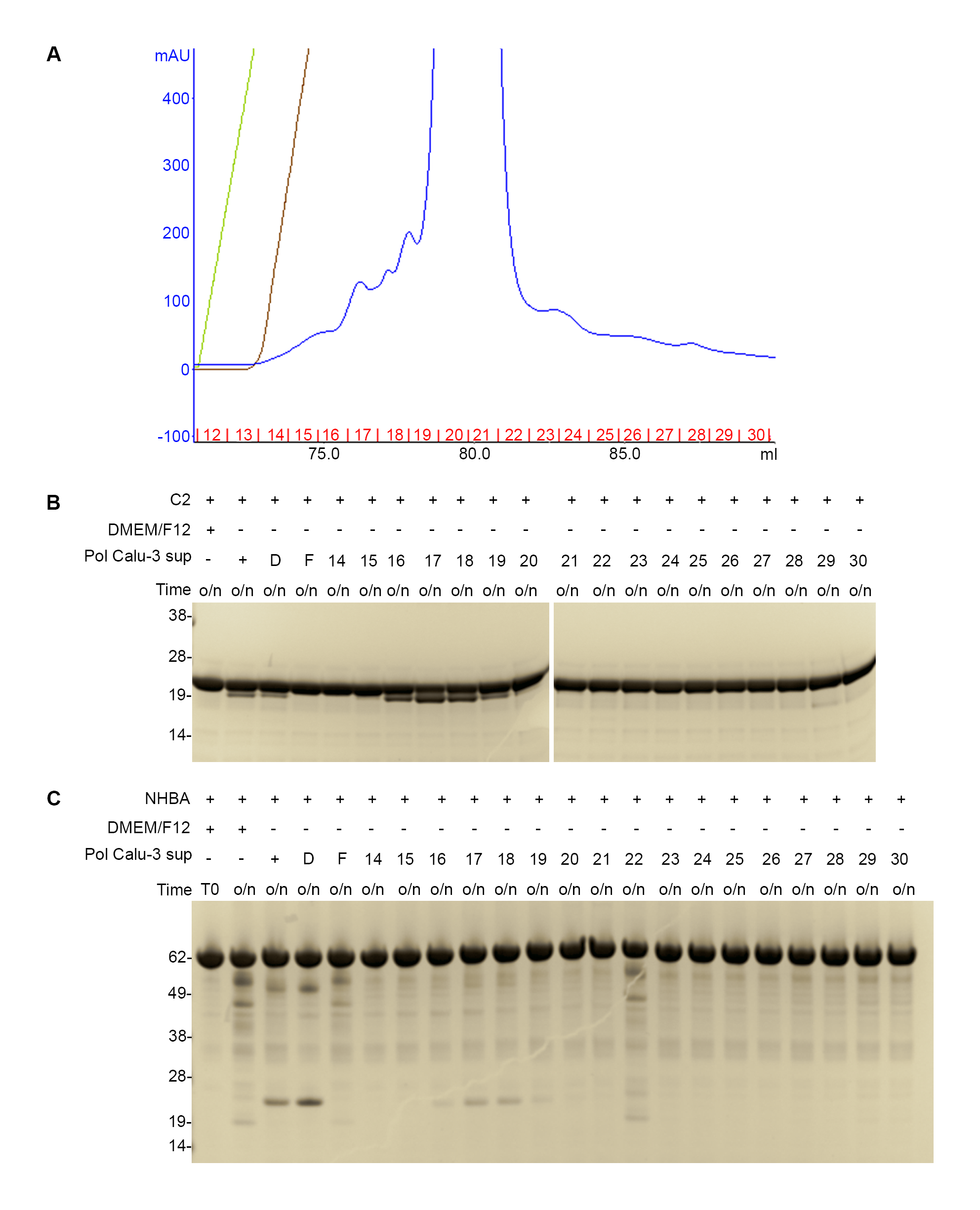

Supplement: S4 Fig — Ion exchange chromatography of polarized Calu-3 cell supernatant A) Chromatogram shows elution of fractions (in red), protein absorbance at 280nm (in blue), salt concentration (in green) and conductivity (in brown). B and C) SDS-PAGE analysis of each eluted fraction incubated overnight (o/n) with 5 μM of recombinant C2-fragment (B) or with 5 μM of recombinant NHBA full length protein (C). Proteins were stained with blue coomassie. (TIF) [file pone.0194662.s004.tif]

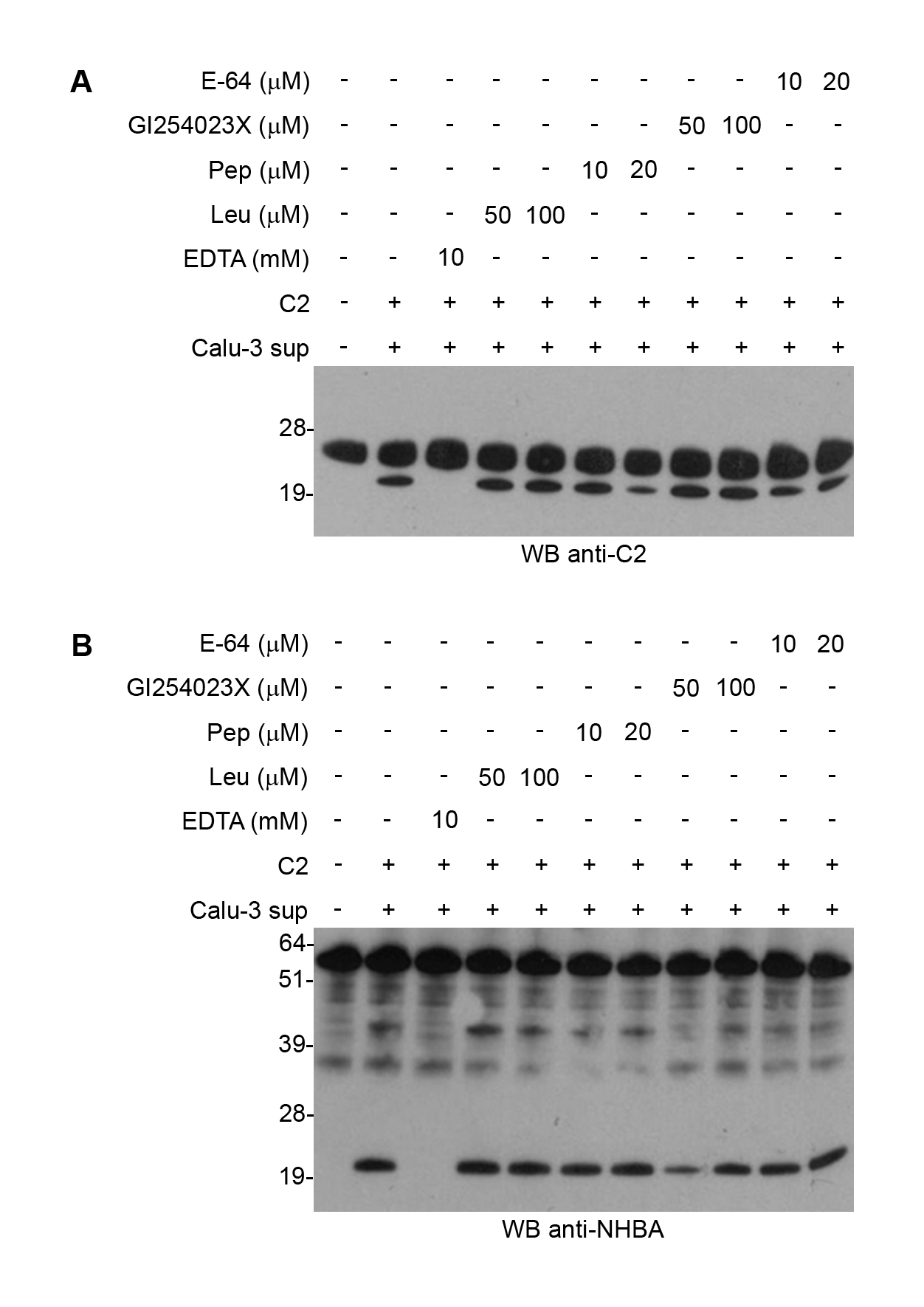

Supplement: S5 Fig — Western blot analysis of recombinant C2 fragment (A) or NHBA full-length protein (B) incubated for 2 hours with Calu-3 cell supernatants that were pre-treated or not with protease inhibitors for 30 minutes. Protease inhibitors tested: EDTA, Leupeptin (Leu), Pepstatine A (Pep), GI254023X and E-64. Polyclonal mouse sera against C2 fragment (A) or against NHBA full-length protein (B) were used for blotting the membranes. (TIF) [file pone.0194662.s005.tif]

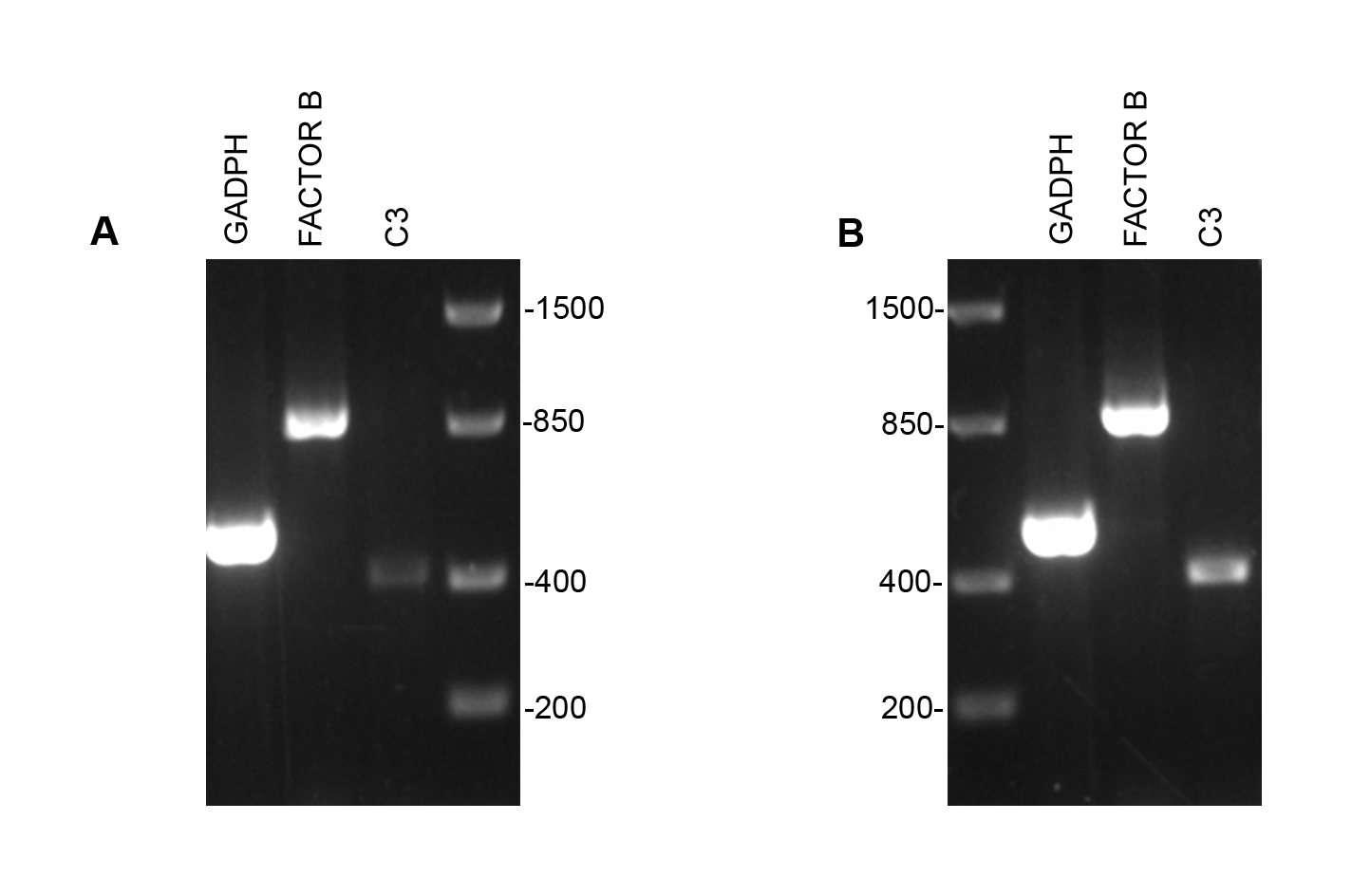

Supplement: S6 Fig — Agarose gel electrophoresis analysis of expression of human CFB and C3 genes in Calu-3 cells (A) and polarized Calu-3 cells (B). GAPDH was used as internal positive control. Total RNA was isolated from epithelial cells, retro transcribed with oligo(dT) and cDNA were used as templates for PCR amplification. For each gene analyzed, specific oligonucleotides amplified part of the mRNA (GAPDH: 518 nt; CFB: 885 nt; C3: 408 nt). (TIF) [file pone.0194662.s006.tif]

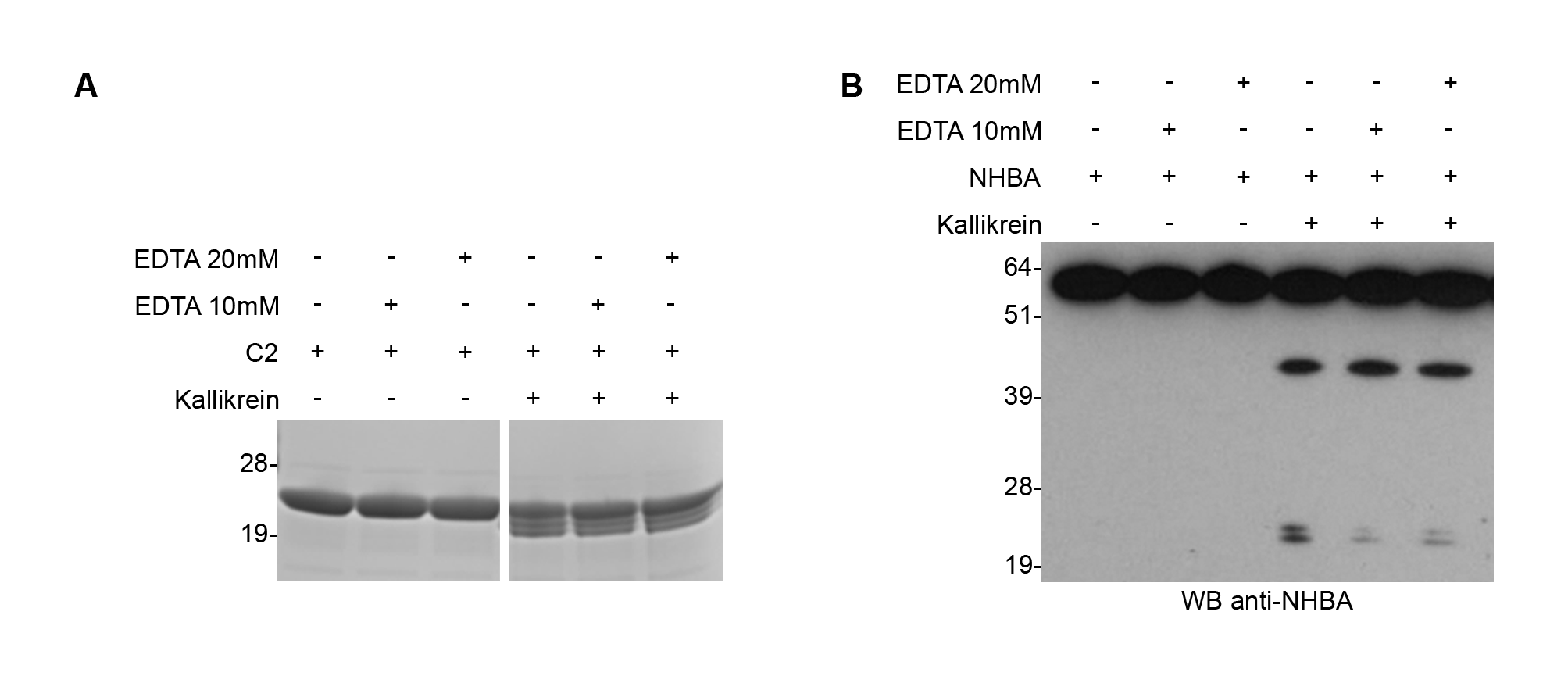

Supplement: S7 Fig — A) SDS-PAGE analysis of recombinant C2 fragment incubated overnight with plasma-purified kallikrein pre-treated or not with EDTA. Proteins were stained with blue coomassie. B) Western blot analysis of recombinant NHBA full-length protein incubated overnight with plasma-purified kallikrein pre-treated or not with EDTA. Polyclonal mouse sera against NHBA full-length protein were used for blotting the membrane. (TIF) [file pone.0194662.s007.tif]
