# Supplementary material for: Neisseria Heparin Binding Antigen is targeted by the human alternative pathway C3-convertase
Source: PLoS One. 2018 Mar 26;13(3):e0194662. doi: 10.1371/journal.pone.0194662 (PMC5868813; doi:10.1371/journal.pone.0194662)
Supplement: S1 Table — (PDF) [file pone.0194662.s008.pdf]

S5 Table. List of proteins contained in selected fractions of Calu-3 cell supernatants identified by mass s

Protein ID	Accession	-10lgP	Coverage (%)	#Peptides	#Unique
1	sp|P21333|FLNA_HUMAN	341.73	36	86	79
2	sp|P21333-2|FLNA_HUMAN	341.73	36	86	79
3	tr|Q5HY54|Q5HY54_HUMAN	341.73	37	86	79
4	sp|P01024|CO3_HUMAN	324.26	38	60	60
5	sp|Q15149-4|PLEC_HUMAN	322.95	29	132	132
6	sp|P35527|K1C9_HUMAN	285.47	45	25	24
7	sp|P01009|A1AT_HUMAN	275.99	52	39	39
8	sp|O75369-8|FLNB_HUMAN	266.52	22	49	43
9	sp|O75369|FLNB_HUMAN	266.52	22	49	43
10	sp|O75369-9|FLNB_HUMAN	266.52	22	49	43
11	sp|O75369-2|FLNB_HUMAN	266.52	22	49	43
12	sp|P01011|AACT_HUMAN	261.68	40	27	25
13	sp|P20810-6|ICAL_HUMAN	256.42	32	19	19
14	sp|P20810-7|ICAL_HUMAN	256.42	33	19	19
15	tr|E7ESM9|E7ESM9_HUMAN	256.42	33	19	19
16	tr|B7Z468|B7Z468_HUMAN	256.42	33	19	19
17	sp|P20810-9|ICAL_HUMAN	256.42	33	19	19
18	sp|P05787-2|K2C8_HUMAN	250.04	56	37	28
19	sp|P05787|K2C8_HUMAN	250.04	59	37	28
20	sp|P08107|HSP71_HUMAN	247.37	32	28	12
21	sp|P08107-2|HSP71_HUMAN	247.37	35	28	12
22	sp|P15311|EZRI_HUMAN	244.67	47	37	29
23	sp|P11142|HSP7C_HUMAN	242.10	40	33	23
24	sp|O75326|SEM7A_HUMAN	230.44	35	24	24
25	sp|Q13753|LAMC2_HUMAN	229.82	25	28	28
26	sp|Q6UX06|OLFM4_HUMAN	228.58	25	17	17
27	sp|P10451|OSTP_HUMAN	224.19	38	10	10
28	sp|P10451-5|OSTP_HUMAN	224.19	39	10	10
29	sp|P10451-2|OSTP_HUMAN	224.19	39	10	10
30	sp|P10451-4|OSTP_HUMAN	224.19	40	10	10
31	sp|P10451-3|OSTP_HUMAN	224.19	41	10	10
32	sp|Q13751|LAMB3_HUMAN	223.81	20	18	17
33	sp|P04264|K2C1_HUMAN	223.21	46	32	25
34	sp|P06396-4|GELS_HUMAN	219.94	27	16	16
35	sp|P06396-2|GELS_HUMAN	219.94	28	16	16
36	sp|P06396|GELS_HUMAN	219.94	26	16	16
37	sp|P06396-3|GELS_HUMAN	219.94	27	16	16
38	tr|S4R3V8|S4R3V8_HUMAN	219.10	40	22	22
39	tr|B4DKL4|B4DKL4_HUMAN	219.10	40	22	22
40	sp|Q86X29-5|LSR_HUMAN	219.10	34	22	22
41	sp|Q86X29|LSR_HUMAN	219.10	31	22	22
42	sp|Q86X29-4|LSR_HUMAN	219.10	32	22	22
43	sp|P08727|K1C19_HUMAN	218.20	54	24	16
44	sp|Q13740|CD166_HUMAN	217.69	25	19	19
45	sp|Q13740-2|CD166_HUMAN	217.69	26	19	19
46	sp|P35908|K22E_HUMAN	217.51	42	25	17
47	sp|P00751|CFAB_HUMAN	216.72	31	28	28
48	tr|B4E1Z4|B4E1Z4_HUMAN	216.72	19	28	28

49	sp|P05783|K1C18_HUMAN	216.09	43	21	19
50	sp|Q16787|LAMA3_HUMAN	215.57	8	25	25
51	sp|O00391|QSOX1_HUMAN	212.22	26	20	20
52	sp|Q9UKY7|CDV3_HUMAN	211.32	75	10	10
53	sp|P11717|MPRI_HUMAN	210.44	10	25	25
54	sp|P07195|LDHB_HUMAN	208.93	32	14	14
55	sp|P60709|ACTB_HUMAN	206.53	39	15	5
56	sp|P63261|ACTG_HUMAN	206.53	39	15	5
57	sp|P14618|KPYM_HUMAN	204.83	33	15	15
58	sp|P13645|K1C10_HUMAN	203.09	29	19	15
59	sp|P27816|MAP4_HUMAN	199.80	22	20	20
60	tr|E7EVA0|E7EVA0_HUMAN	199.80	11	20	20
61	sp|Q09666|AHNK_HUMAN	199.50	6	24	24
62	sp|P04075-2|ALDOA_HUMAN	197.51	44	18	17
63	tr|J3KPS3|J3KPS3_HUMAN	197.51	51	18	17
64	sp|P04075|ALDOA_HUMAN	197.51	51	18	17
65	sp|P19021-6|AMD_HUMAN	196.01	17	19	19
66	sp|P19021-5|AMD_HUMAN	196.01	17	19	19
67	sp|P19021|AMD_HUMAN	196.01	17	19	19
68	sp|P19021-2|AMD_HUMAN	195.22	19	19	19
69	sp|Q14118|DAG1_HUMAN	195.99	16	14	14
70	sp|P10586|PTPRF_HUMAN	194.99	12	20	19
71	sp|P10586-2|PTPRF_HUMAN	194.99	12	20	19
72	sp|O00469-2|PLOD2_HUMAN	194.99	21	13	12
73	sp|O00469|PLOD2_HUMAN	194.99	22	13	12
74	sp|P18206|VINC_HUMAN	193.23	16	16	16
75	sp|P18206-2|VINC_HUMAN	193.23	17	16	16
76	sp|P05362|ICAM1_HUMAN	188.47	29	14	14
77	sp|P12109|CO6A1_HUMAN	188.46	14	13	13
78	sp|Q13308-5|PTK7_HUMAN	188.41	19	14	13
79	sp|Q13308-2|PTK7_HUMAN	188.41	15	14	13
80	sp|Q13308-6|PTK7_HUMAN	188.41	14	14	13
81	sp|Q13308|PTK7_HUMAN	188.41	14	14	13
82	sp|P08729|K2C7_HUMAN	187.08	45	25	16
83	sp|P06733|ENOA_HUMAN	186.88	30	14	14
84	sp|Q14247|SRC8_HUMAN	186.72	28	15	15
85	tr|J9JID7|J9JID7_HUMAN	185.50	36	23	20
86	sp|Q03252|LMNB2_HUMAN	185.50	37	23	20
87	tr|D6RBV2|D6RBV2_HUMAN	185.45	27	9	9
88	sp|Q12907|LMAN2_HUMAN	185.45	25	9	9
89	sp|P26038|MOES_HUMAN	183.66	29	16	8
90	sp|P27487|DPP4_HUMAN	181.89	21	15	15
91	sp|Q9UK76|HN1_HUMAN	181.33	65	9	9
92	sp|P13639|EF2_HUMAN	180.83	17	14	14
93	sp|P08603|CFAH_HUMAN	180.57	11	13	13
94	sp|O00468-3|AGRIN_HUMAN	180.46	9	17	17
95	sp|O00468-6|AGRIN_HUMAN	180.46	9	17	17
96	sp|P08581|MET_HUMAN	180.27	12	16	16
97	sp|P02533|K1C14_HUMAN	179.91	26	12	3
98	sp|P04626|ERBB2_HUMAN	179.58	11	14	13

99	sp|Q86UP2|KTN1_HUMAN	179.29	16	18	18
100	sp|Q86UP2-3|KTN1_HUMAN	179.29	17	18	18
101	sp|Q86UP2-4|KTN1_HUMAN	179.29	17	18	18
102	sp|Q86UP2-2|KTN1_HUMAN	179.29	17	18	18
103	sp|O75223|GGCT_HUMAN	178.03	51	12	12
104	sp|Q06830|PRDX1_HUMAN	177.85	48	12	11
105	sp|P43490|NAMPT_HUMAN	176.32	20	10	10
106	sp|P28838-2|AMPL_HUMAN	176.03	33	13	13
107	sp|P28838|AMPL_HUMAN	176.03	31	13	13
108	sp|Q02809|PLOD1_HUMAN	175.41	22	12	11
109	sp|Q6YHK3|CD109_HUMAN	174.10	11	19	17
110	sp|Q6YHK3-4|CD109_HUMAN	174.10	11	19	17
111	sp|P18669|PGAM1_HUMAN	172.00	26	6	6
112	sp|P55011|S12A2_HUMAN	170.85	11	7	7
113	sp|P55011-3|S12A2_HUMAN	170.85	11	7	7
114	tr|G3XAL9|G3XAL9_HUMAN	170.85	12	7	7
115	sp|P32004-3|L1CAM_HUMAN	169.90	13	16	16
116	tr|F5H025|F5H025_HUMAN	169.90	13	16	16
117	sp|P32004|L1CAM_HUMAN	169.90	13	16	16
118	tr|F5H1H0|F5H1H0_HUMAN	169.90	13	16	16
119	sp|P32004-2|L1CAM_HUMAN	169.90	13	16	16
120	sp|P12955-2|PEPD_HUMAN	169.46	20	10	10
121	sp|P12955|PEPD_HUMAN	169.46	19	10	10
122	sp|P05120|PAI2_HUMAN	168.67	27	11	11
123	sp|P20061|TCO1_HUMAN	167.42	18	8	8
124	sp|P14866|HNRPL_HUMAN	167.06	18	6	6
125	sp|Q7Z7M0|MEGF8_HUMAN	166.56	5	11	11
126	sp|P50395-2|GDIB_HUMAN	165.09	30	11	10
127	tr|E7EU23|E7EU23_HUMAN	165.09	27	11	10
128	sp|P50395|GDIB_HUMAN	165.09	27	11	10
129	sp|O95433|AHSA1_HUMAN	164.49	30	10	10
130	sp|P11047|LAMC1_HUMAN	163.69	9	13	13
131	sp|P30740|ILEU_HUMAN	163.05	29	10	10
132	sp|Q96KP4|CNDP2_HUMAN	161.94	26	10	10
133	sp|O14745|NHRF1_HUMAN	161.78	34	8	8
134	sp|O15031|PLXB2_HUMAN	161.03	7	10	10
135	sp|Q9HB71|CYBP_HUMAN	160.86	32	8	8
136	sp|P98160|PGBM_HUMAN	158.23	2	9	9
137	sp|P19338|NUCL_HUMAN	158.03	16	9	9
138	tr|E9PBF6|E9PBF6_HUMAN	158.00	54	20	17
139	sp|P20700|LMNB1_HUMAN	158.00	36	20	17
140	sp|Q9GZP0|PDGFD_HUMAN	157.53	24	10	10
141	sp|Q16881-6|TRXR1_HUMAN	157.05	11	7	7
142	tr|E7EW10|E7EW10_HUMAN	157.05	11	7	7
143	tr|E9PIR7|E9PIR7_HUMAN	157.05	15	7	7
144	tr|B2R5P6|B2R5P6_HUMAN	157.05	14	7	7
145	sp|Q16881-5|TRXR1_HUMAN	157.05	14	7	7
146	tr|E2QRB9|E2QRB9_HUMAN	157.05	13	7	7
147	sp|Q16881-2|TRXR1_HUMAN	157.05	13	7	7
148	tr|B7Z2S5|B7Z2S5_HUMAN	157.05	13	7	7

149	sp|Q16881-4|TRXR1_HUMAN	157.05	13	7	7
150	tr|E9PNQ6|E9PNQ6_HUMAN	157.05	12	7	7
151	tr|E7ESI6|E7ESI6_HUMAN	157.05	12	7	7
152	sp|Q16881-3|TRXR1_HUMAN	157.05	12	7	7
153	sp|Q16881|TRXR1_HUMAN	157.05	11	7	7
154	sp|Q12882|DPYD_HUMAN	156.20	14	11	11
155	sp|Q96NY8|PVRL4_HUMAN	155.28	21	9	9
156	sp|P38646|GRP75_HUMAN	155.17	24	12	10
157	sp|P61978-3|HNRPK_HUMAN	155.05	24	11	11
158	sp|P61978-2|HNRPK_HUMAN	155.05	23	11	11
159	sp|P61978|HNRPK_HUMAN	155.05	23	11	11
160	sp|P17096|HMGA1_HUMAN	154.80	49	5	5
161	sp|P22626|ROA2_HUMAN	152.83	26	10	8
162	sp|Q9P258|RCC2_HUMAN	152.15	19	9	9
163	sp|Q14126|DSG2_HUMAN	151.37	10	9	9
164	sp|P09960|LKHA4_HUMAN	149.67	13	6	6
165	sp|P10909-5|CLUS_HUMAN	148.93	20	11	11
166	sp|P10909|CLUS_HUMAN	148.93	21	11	11
167	sp|P10909-2|CLUS_HUMAN	148.93	19	11	11
168	tr|A0AVL1|A0AVL1_HUMAN	148.43	6	7	7
169	tr|F8WC54|F8WC54_HUMAN	148.43	7	7	7
170	sp|Q13443-2|ADAM9_HUMAN	148.43	7	7	7
171	sp|Q13443|ADAM9_HUMAN	148.43	5	7	7
172	sp|P17936|IBP3_HUMAN	147.22	32	8	8
173	sp|P17936-2|IBP3_HUMAN	147.22	32	8	8
174	sp|P11021|GRP78_HUMAN	147.11	8	6	3
175	sp|P35613-2|BASI_HUMAN	146.40	25	5	5
176	sp|Q15582|BGH3_HUMAN	145.54	11	9	9
177	tr|G8JLA8|G8JLA8_HUMAN	145.54	11	9	9
178	sp|Q08345-2|DDR1_HUMAN	144.63	10	8	8
179	sp|Q08345-6|DDR1_HUMAN	144.63	10	8	8
180	sp|Q08345-5|DDR1_HUMAN	144.63	9	8	8
181	sp|Q08345|DDR1_HUMAN	144.63	9	8	8
182	sp|P07339|CATD_HUMAN	144.34	21	9	9
183	tr|B4E022|B4E022_HUMAN	143.95	20	9	9
184	sp|P29401|TKT_HUMAN	143.95	18	9	9
185	sp|P29401-2|TKT_HUMAN	143.95	18	9	9
186	sp|P13647|K2C5_HUMAN	142.49	17	11	3
187	tr|J3KTF8|J3KTF8_HUMAN	141.03	31	7	6
188	sp|P52565|GDIR1_HUMAN	141.03	29	7	6
189	tr|J3QQX2|J3QQX2_HUMAN	141.03	25	7	6
190	sp|Q01082|SPTB2_HUMAN	140.95	3	5	5
191	sp|P16949|STMN1_HUMAN	139.30	38	8	8
192	sp|P16949-2|STMN1_HUMAN	139.30	32	8	8
193	sp|Q8WVQ1-3|CANT1_HUMAN	139.19	30	9	9
194	sp|Q8WVQ1|CANT1_HUMAN	139.19	26	9	9
195	sp|P54727|RD23B_HUMAN	138.17	21	6	6
196	sp|O75882-2|ATRN_HUMAN	138.14	5	5	5
197	sp|O75882|ATRN_HUMAN	138.14	4	5	5
198	sp|P48637-2|GSHB_HUMAN	137.80	21	8	8

199	sp|P48637|GSHB_HUMAN	137.80	16	8	8
200	tr|E7ESM2|E7ESM2_HUMAN	137.40	14	5	5
201	sp|P52888|THOP1_HUMAN	136.99	13	8	7
202	sp|Q96HE7|ERO1A_HUMAN	136.07	10	6	6
203	sp|O75436|VP26A_HUMAN	136.04	25	6	6
204	sp|Q9NQS3-2|PVRL3_HUMAN	135.20	11	3	3
205	sp|Q9NQS3-3|PVRL3_HUMAN	135.20	8	3	3
206	sp|Q9NQS3|PVRL3_HUMAN	135.20	7	3	3
207	sp|Q14103-3|HNRPD_HUMAN	134.36	23	7	6
208	sp|Q14103|HNRPD_HUMAN	134.36	20	7	6
209	tr|Q30118|Q30118_HUMAN	134.31	24	4	4
210	sp|P01903|DRA_HUMAN	134.31	21	4	4
211	sp|P42167|LAP2B_HUMAN	133.94	15	6	6
212	sp|Q13263-2|TIF1B_HUMAN	133.90	14	9	9
213	sp|Q13263|TIF1B_HUMAN	133.90	13	9	9
214	tr|B1AKC9|B1AKC9_HUMAN	133.55	6	6	6
215	sp|P29323-2|EPHB2_HUMAN	133.55	6	6	6
216	sp|P29323-3|EPHB2_HUMAN	133.55	6	6	6
217	sp|P29323|EPHB2_HUMAN	133.55	6	6	6
218	sp|P31948|STIP1_HUMAN	133.49	15	8	8
219	sp|O00754|MA2B1_HUMAN	132.91	7	6	6
220	sp|O00754-2|MA2B1_HUMAN	132.91	7	6	6
221	tr|B1AK87|B1AK87_HUMAN	132.55	20	5	5
222	sp|P47756-2|CAPZB_HUMAN	132.55	19	5	5
223	tr|B1AK88|B1AK88_HUMAN	132.55	17	5	5
224	sp|P13760|2B14_HUMAN	132.29	20	5	3
225	sp|P00558|PGK1_HUMAN	131.78	15	6	6
226	sp|Q92804-2|RBP56_HUMAN	131.75	14	6	4
227	sp|Q92804|RBP56_HUMAN	131.75	14	6	4
228	sp|Q05639|EF1A2_HUMAN	131.45	12	5	5
229	sp|P68104|EF1A1_HUMAN	131.45	12	5	5
230	sp|Q5VTE0|EF1A3_HUMAN	131.45	12	5	5
231	sp|P61604|CH10_HUMAN	131.25	69	7	6
232	sp|Q8NFZ8|CADM4_HUMAN	131.21	15	5	5
233	sp|P10253|LYAG_HUMAN	130.96	9	6	6
234	sp|O95831-3|AIFM1_HUMAN	130.67	12	6	6
235	sp|O95831|AIFM1_HUMAN	130.67	12	6	6
236	sp|O94788|AL1A2_HUMAN	130.60	14	6	5
237	sp|P62937|PPIA_HUMAN	130.03	28	7	7
238	tr|H7C3P4|H7C3P4_HUMAN	129.65	12	6	6
239	tr|B4DYH8|B4DYH8_HUMAN	129.65	11	6	6
240	sp|P15586|GNS_HUMAN	129.65	11	6	6
241	tr|F6S8M0|F6S8M0_HUMAN	129.65	10	6	6
242	sp|Q8WW12|PCNP_HUMAN	129.48	36	5	5
243	sp|P08779|K1C16_HUMAN	128.57	23	9	4
244	tr|B4DHG0|B4DHG0_HUMAN	127.42	17	7	7
245	tr|E9PEX6|E9PEX6_HUMAN	127.42	15	7	7
246	sp|P09622|DLDH_HUMAN	127.42	14	7	7
247	tr|K7EMN2|K7EMN2_HUMAN	127.25	32	5	5
248	tr|K7EM49|K7EM49_HUMAN	127.25	25	5	5

249	tr|K7EPF6|K7EPF6_HUMAN	127.25	20	5	5
250	tr|F5H7U0|F5H7U0_HUMAN	127.25	11	5	5
251	tr|B4DQJ8|B4DQJ8_HUMAN	127.25	11	5	5
252	sp|P52209|6PGD_HUMAN	127.25	11	5	5
253	sp|Q16706|MA2A1_HUMAN	127.25	9	9	9
254	sp|P02765|FETUA_HUMAN	127.20	9	6	6
255	sp|Q9UBR2|CATZ_HUMAN	126.92	21	6	6
256	sp|P43121|MUC18_HUMAN	126.74	11	6	6
257	sp|Q9Y6N7-6|ROBO1_HUMAN	125.55	7	8	8
258	sp|Q9Y6N7-5|ROBO1_HUMAN	125.55	6	8	8
259	sp|Q9Y6N7-4|ROBO1_HUMAN	125.55	6	8	8
260	sp|Q9Y6N7-3|ROBO1_HUMAN	125.55	6	8	8
261	sp|Q9Y6N7|ROBO1_HUMAN	125.55	6	8	8
262	sp|Q9Y6N7-2|ROBO1_HUMAN	125.55	6	8	8
263	tr|D6R9P3|D6R9P3_HUMAN	121.66	22	7	7
264	sp|Q99729-3|ROAA_HUMAN	121.66	22	7	7
265	tr|D6RD18|D6RD18_HUMAN	121.66	22	7	7
266	tr|D6RBZ0|D6RBZ0_HUMAN	121.66	19	7	7
267	sp|Q99729-2|ROAA_HUMAN	121.66	19	7	7
268	sp|Q01518-2|CAP1_HUMAN	121.41	18	8	8
269	sp|Q01518|CAP1_HUMAN	121.41	18	8	8
270	sp|O75083|WDR1_HUMAN	121.16	13	6	6
271	sp|P54802|ANAG_HUMAN	120.61	9	4	4
272	sp|P04406|G3P_HUMAN	118.88	19	5	5
273	sp|P09651-3|ROA1_HUMAN	118.60	24	6	4
274	sp|P09651|ROA1_HUMAN	118.60	17	6	4
275	tr|F8W6I7|F8W6I7_HUMAN	118.60	21	6	4
276	sp|P09651-2|ROA1_HUMAN	118.60	20	6	4
277	sp|P15151-3|PVR_HUMAN	118.55	18	5	5
278	sp|P15151-2|PVR_HUMAN	118.55	17	5	5
279	sp|P15151-4|PVR_HUMAN	118.55	16	5	5
280	sp|P15151|PVR_HUMAN	118.55	15	5	5
281	sp|P13646|K1C13_HUMAN	118.46	17	8	3
282	tr|E9PPJ0|E9PPJ0_HUMAN	118.03	6	4	4
283	sp|Q13435|SF3B2_HUMAN	118.03	6	4	4
284	sp|Q9GZM7-3|TINAL_HUMAN	116.87	14	5	5
285	sp|Q9GZM7|TINAL_HUMAN	116.87	13	5	5
286	sp|Q9BRA2|TXD17_HUMAN	116.36	38	4	4
287	sp|O60506-4|HNRPQ_HUMAN	116.29	13	4	4
288	sp|O60506-3|HNRPQ_HUMAN	116.29	13	4	4
289	sp|O60506-2|HNRPQ_HUMAN	116.29	12	4	4
290	sp|O60506|HNRPQ_HUMAN	116.29	11	4	4
291	tr|K7EQ71|K7EQ71_HUMAN	115.78	6	5	5
292	tr|K7EKI8|K7EKI8_HUMAN	115.78	4	5	5
293	sp|O60437|PEPL_HUMAN	115.78	4	5	5
294	sp|P12429|ANXA3_HUMAN	115.63	23	6	6
295	tr|B4DZI8|B4DZI8_HUMAN	115.49	9	6	6
296	sp|P35606|COPB2_HUMAN	115.49	9	6	6
297	sp|O14979|HNRDL_HUMAN	114.38	10	5	4
298	sp|O14979-3|HNRDL_HUMAN	114.38	18	5	4

299	sp|O14979-2|HNRDL_HUMAN	114.38	14	5	4
300	sp|P02768|ALBU_HUMAN	114.26	8	6	6
301	sp|Q13541|4EBP1_HUMAN	114.20	36	4	4
302	sp|O94760|DDAH1_HUMAN	114.11	21	6	6
303	sp|P13688-2|CEAM1_HUMAN	113.72	11	3	3
304	sp|P13688-8|CEAM1_HUMAN	113.72	10	3	3
305	sp|P13688-10|CEAM1_HUMAN	113.72	10	3	3
306	sp|P13688|CEAM1_HUMAN	113.72	9	3	3
307	tr|E7EMM4|E7EMM4_HUMAN	113.52	14	6	6
308	sp|Q13510|ASAH1_HUMAN	113.52	13	6	6
309	sp|Q13510-2|ASAH1_HUMAN	113.52	13	6	6
310	tr|I3L397|I3L397_HUMAN	112.30	14	3	3
311	sp|P63241|IF5A1_HUMAN	112.30	14	3	3
312	sp|P63241-2|IF5A1_HUMAN	112.30	11	3	3
313	tr|I3L504|I3L504_HUMAN	112.30	11	3	3
314	tr|F8WCJ1|F8WCJ1_HUMAN	108.34	20	3	3
315	tr|C9J7B5|C9J7B5_HUMAN	108.34	19	3	3
316	tr|C9J4W5|C9J4W5_HUMAN	108.34	18	3	3
317	sp|Q9GZV4|IF5A2_HUMAN	108.34	14	3	3
318	sp|P10599|THIO_HUMAN	111.48	43	4	4
319	sp|P50453|SPB9_HUMAN	111.30	11	4	4
320	sp|O60716-15|CTND1_HUMAN	110.99	7	5	5
321	sp|O60716-16|CTND1_HUMAN	110.99	7	5	5
322	sp|O60716-11|CTND1_HUMAN	110.99	7	5	5
323	sp|O60716-13|CTND1_HUMAN	110.99	7	5	5
324	sp|O60716-12|CTND1_HUMAN	110.99	7	5	5
325	sp|O60716-14|CTND1_HUMAN	110.99	7	5	5
326	sp|O60716-10|CTND1_HUMAN	110.99	7	5	5
327	sp|O60716-9|CTND1_HUMAN	110.99	7	5	5
328	sp|O60716-8|CTND1_HUMAN	110.99	7	5	5
329	sp|O60716-7|CTND1_HUMAN	110.99	7	5	5
330	sp|O60716-3|CTND1_HUMAN	110.99	6	5	5
331	tr|C9JZR2|C9JZR2_HUMAN	110.99	7	5	5
332	sp|O60716-5|CTND1_HUMAN	110.99	7	5	5
333	sp|O60716-6|CTND1_HUMAN	110.99	6	5	5
334	sp|O60716-4|CTND1_HUMAN	110.99	6	5	5
335	sp|O60716-2|CTND1_HUMAN	110.99	6	5	5
336	sp|O60716|CTND1_HUMAN	110.99	6	5	5
337	tr|F5H335|F5H335_HUMAN	110.85	3	6	6
338	sp|Q14152|EIF3A_HUMAN	110.85	3	6	6
339	sp|P35237|SPB6_HUMAN	110.67	10	3	3
340	sp|P61956|SUMO2_HUMAN	110.58	34	4	4
341	sp|P31150|GDIA_HUMAN	110.13	15	6	5
342	sp|O14672|ADA10_HUMAN	109.97	9	4	4
343	sp|Q15181|IPYR_HUMAN	109.84	13	3	3
344	sp|P53396-2|ACLY_HUMAN	109.44	4	5	5
345	sp|P53396|ACLY_HUMAN	109.44	4	5	5
346	sp|P46109|CRKL_HUMAN	109.42	18	5	5
347	tr|C9JUT4|C9JUT4_HUMAN	106.83	13	4	4
348	sp|Q93052|LPP_HUMAN	106.83	9	4	4

349	sp|Q9Y5Y6|ST14_HUMAN	106.67	7	6	6
350	sp|O60449|LY75_HUMAN	106.60	3	4	4
351	sp|O60449-3|LY75_HUMAN	106.60	3	4	4
352	sp|O60449-2|LY75_HUMAN	106.60	3	4	4
353	sp|P07711|CATL1_HUMAN	106.53	13	4	4
354	sp|P01019|ANGT_HUMAN	106.40	8	5	5
355	sp|P00338|LDHA_HUMAN	106.24	13	4	4
356	sp|P00338-3|LDHA_HUMAN	106.24	12	4	4
357	sp|Q16555-2|DPYL2_HUMAN	106.17	13	5	4
358	sp|Q16555|DPYL2_HUMAN	106.17	12	5	4
359	sp|P32119|PRDX2_HUMAN	106.15	27	6	5
360	tr|E9PBJ4|E9PBJ4_HUMAN	105.02	22	3	3
361	sp|P07437|TBB5_HUMAN	105.02	12	3	3
362	tr|Q5JP53|Q5JP53_HUMAN	105.02	13	3	3
363	sp|Q9HCN8|SDF2L_HUMAN	104.70	30	3	3
364	sp|P17174|AATC_HUMAN	104.58	9	4	4
365	tr|B7Z7E9|B7Z7E9_HUMAN	99.43	9	4	4
366	sp|Q03154-2|ACY1_HUMAN	104.29	17	6	6
367	sp|Q03154-4|ACY1_HUMAN	104.29	16	6	6
368	sp|Q03154|ACY1_HUMAN	104.29	14	6	6
369	tr|B4DNW0|B4DNW0_HUMAN	104.29	12	6	6
370	sp|Q8NC51-2|PAIRB_HUMAN	103.83	10	4	4
371	sp|Q8NC51|PAIRB_HUMAN	103.83	10	4	4
372	tr|H0Y2Y8|H0Y2Y8_HUMAN	103.76	12	5	5
373	sp|Q15942|ZYX_HUMAN	103.76	11	5	5
374	sp|P01833|PIGR_HUMAN	103.65	8	4	4
375	sp|P40121-2|CAPG_HUMAN	103.61	14	5	5
376	sp|P40121|CAPG_HUMAN	103.61	13	5	5
377	sp|Q7KZF4|SND1_HUMAN	103.06	7	5	5
378	sp|P02788-2|TRFL_HUMAN	102.94	7	5	4
379	tr|E7EQB2|E7EQB2_HUMAN	102.94	7	5	4
380	tr|E7ER44|E7ER44_HUMAN	102.94	6	5	4
381	sp|P02788|TRFL_HUMAN	102.94	6	5	4
382	sp|O75475-3|PSIP1_HUMAN	102.94	12	5	5
383	sp|O75475-2|PSIP1_HUMAN	102.94	12	5	5
384	sp|O75475|PSIP1_HUMAN	102.94	8	5	5
385	sp|Q96IZ0|PAWR_HUMAN	102.62	15	4	4
386	tr|H3BVD9|H3BVD9_HUMAN	102.48	12	5	5
387	sp|O43278-2|SPIT1_HUMAN	102.48	11	5	5
388	sp|O43278|SPIT1_HUMAN	102.48	11	5	5
389	sp|Q9Y2V2|CHSP1_HUMAN	102.36	32	3	3
390	sp|Q16651|PRSS8_HUMAN	102.11	16	4	4
391	sp|P53634|CATC_HUMAN	102.04	13	6	6
392	tr|Q5T123|Q5T123_HUMAN	101.99	34	3	3
393	sp|Q9H299|SH3L3_HUMAN	101.99	32	3	3
394	sp|O95989|NUDT3_HUMAN	101.82	26	3	3
395	tr|Q5T0D2|Q5T0D2_HUMAN	101.47	37	5	5
396	sp|P30085|KCY_HUMAN	101.47	32	5	5
397	sp|Q14195-2|DPYL3_HUMAN	101.41	10	5	4
398	sp|P30040|ERP29_HUMAN	101.31	16	4	4

399	sp|P02766|TTHY_HUMAN	101.12	14	4	4
400	tr|E9PMS6|E9PMS6_HUMAN	101.01	3	4	4
401	sp|P12270|TPR_HUMAN	99.82	3	5	5
402	sp|P52907|CAZA1_HUMAN	99.45	17	4	3
403	sp|Q92692-2|PVRL2_HUMAN	99.18	8	4	4
404	sp|Q92692|PVRL2_HUMAN	99.18	7	4	4
405	tr|M0R1V7|M0R1V7_HUMAN	98.90	38	3	3
406	tr|J3QS39|J3QS39_HUMAN	98.90	26	3	3
407	tr|J3QTR3|J3QTR3_HUMAN	98.90	23	3	3
408	sp|P62987|RL40_HUMAN	98.90	19	3	3
409	sp|P62979|RS27A_HUMAN	98.90	15	3	3
410	tr|F5H747|F5H747_HUMAN	98.90	15	3	3
411	tr|J3QKN0|J3QKN0_HUMAN	98.90	12	3	3
412	tr|J3QLP7|J3QLP7_HUMAN	98.90	11	3	3
413	tr|J3QRK5|J3QRK5_HUMAN	98.90	10	3	3
414	sp|P0CG47|UBB_HUMAN	98.90	10	3	3
415	tr|Q96C32|Q96C32_HUMAN	98.90	8	3	3
416	sp|P0CG48|UBC_HUMAN	98.90	4	3	3
417	sp|P07686|HEXB_HUMAN	98.72	9	5	4
418	sp|Q99523-2|SORT_HUMAN	98.40	6	3	3
419	sp|Q99523|SORT_HUMAN	98.40	5	3	3
420	sp|Q9UKU6|TRHDE_HUMAN	98.15	4	3	3
421	tr|F8W1Q3|F8W1Q3_HUMAN	98.13	7	3	3
422	sp|P43251-2|BTD_HUMAN	98.13	6	3	3
423	sp|P43251-3|BTD_HUMAN	98.13	6	3	3
424	sp|P43251|BTD_HUMAN	98.13	6	3	3
425	sp|P68371|TBB4B_HUMAN	97.93	12	3	3
426	sp|P31942-4|HNRH3_HUMAN	97.83	21	3	3
427	tr|B4DHY1|B4DHY1_HUMAN	97.83	19	3	3
428	sp|P31942-3|HNRH3_HUMAN	97.83	15	3	3
429	sp|P31942-2|HNRH3_HUMAN	97.83	14	3	3
430	sp|P31942|HNRH3_HUMAN	97.83	13	3	3
431	sp|P53999|TCP4_HUMAN	97.25	26	3	3
432	sp|P37837|TALDO_HUMAN	96.80	12	4	4
433	sp|Q12841|FSTL1_HUMAN	96.80	11	4	4
434	sp|P30520|PURA2_HUMAN	96.49	16	5	5
435	sp|Q9BRK5-4|CAB45_HUMAN	96.47	25	4	4
436	tr|G3V1E2|G3V1E2_HUMAN	96.47	20	4	4
437	sp|Q9BRK5-3|CAB45_HUMAN	96.47	20	4	4
438	sp|Q9BRK5-6|CAB45_HUMAN	96.47	15	4	4
439	sp|Q9BRK5|CAB45_HUMAN	96.47	14	4	4
440	tr|E9PBS1|E9PBS1_HUMAN	96.00	10	3	3
441	sp|P22234|PUR6_HUMAN	96.00	10	3	3
442	sp|P22234-2|PUR6_HUMAN	96.00	10	3	3
443	sp|P10809|CH60_HUMAN	95.83	11	6	5
444	sp|P17050|NAGAB_HUMAN	95.76	7	3	3
445	sp|P00367|DHE3_HUMAN	95.07	9	4	4
446	sp|Q13011|ECH1_HUMAN	94.96	12	3	3
447	sp|P61160|ARP2_HUMAN	94.89	14	4	4
448	sp|Q7Z7D3-2|VTCN1_HUMAN	93.97	8	3	3

449	sp|Q7Z7D3-4|VTCN1_HUMAN	93.97	7	3	3
450	tr|Q5T2L0|Q5T2L0_HUMAN	93.97	5	3	3
451	sp|Q7Z7D3|VTCN1_HUMAN	93.97	5	3	3
452	tr|B7Z8J4|B7Z8J4_HUMAN	93.65	25	3	3
453	sp|P30419|NMT1_HUMAN	93.65	7	3	3
454	sp|Q9UM22|EPDR1_HUMAN	93.08	15	3	3
455	tr|A4D1W8|A4D1W8_HUMAN	93.08	10	3	3
456	sp|P15924-3|DESP_HUMAN	92.59	2	4	3
457	sp|P15924|DESP_HUMAN	92.59	1	4	3
458	tr|D6R991|D6R991_HUMAN	92.42	9	3	3
459	tr|D6REM6|D6REM6_HUMAN	92.42	5	3	3
460	sp|P43243|MATR3_HUMAN	92.42	5	3	3
461	tr|A8MXP9|A8MXP9_HUMAN	92.42	4	3	3
462	sp|P02771|FETA_HUMAN	92.35	5	3	3
463	tr|J3KMX3|J3KMX3_HUMAN	92.35	5	3	3
464	sp|P49257|LMAN1_HUMAN	92.26	9	3	3
465	sp|Q9UMX5|NENF_HUMAN	92.22	17	3	3
466	sp|P23246|SFPQ_HUMAN	91.60	11	7	7
467	sp|Q13242|SRSF9_HUMAN	91.51	14	3	3
468	sp|P80188|NGAL_HUMAN	90.99	17	3	3
469	sp|P80188-2|NGAL_HUMAN	90.99	17	3	3
470	tr|X6R8F3|X6R8F3_HUMAN	90.99	16	3	3
471	tr|H9KV70|H9KV70_HUMAN	90.99	16	3	3
472	sp|O75663|TIPRL_HUMAN	90.95	17	4	4
473	sp|P02751-17|FINC_HUMAN	90.91	2	3	3
474	sp|Q9NPH3-5|IL1AP_HUMAN	90.07	4	3	3
475	sp|Q9NPH3-2|IL1AP_HUMAN	86.48	8	3	3
476	sp|Q9NPH3|IL1AP_HUMAN	86.48	5	3	3
477	sp|P26641|EF1G_HUMAN	89.52	7	4	4
478	tr|B4DTG2|B4DTG2_HUMAN	89.52	6	4	4
479	sp|P00491|PNPH_HUMAN	88.72	12	3	3
480	sp|P07355|ANXA2_HUMAN	88.56	22	6	6
481	sp|P07355-2|ANXA2_HUMAN	88.56	21	6	6
482	sp|P07996|TSP1_HUMAN	88.40	3	3	3
483	tr|E7ETE2|E7ETE2_HUMAN	88.33	11	3	3
484	tr|B4DDD8|B4DDD8_HUMAN	88.33	7	3	3
485	sp|P12081-4|SYHC_HUMAN	88.33	6	3	3
486	sp|P12081|SYHC_HUMAN	88.33	6	3	3
487	tr|B4E1C5|B4E1C5_HUMAN	88.33	8	3	3
488	sp|P12081-3|SYHC_HUMAN	88.33	7	3	3
489	sp|P12081-2|SYHC_HUMAN	88.33	7	3	3
490	sp|Q12805-5|FBLN3_HUMAN	88.32	7	3	3
491	sp|Q12805-2|FBLN3_HUMAN	88.32	5	3	3
492	sp|Q12805-4|FBLN3_HUMAN	88.32	5	3	3
493	sp|Q12805-3|FBLN3_HUMAN	88.32	5	3	3
494	sp|Q12805|FBLN3_HUMAN	88.32	5	3	3
495	sp|P01008|ANT3_HUMAN	87.04	10	6	5
496	tr|G3V0E5|G3V0E5_HUMAN	85.61	6	3	3
497	sp|P02786|TFR1_HUMAN	85.61	5	3	3
498	tr|Q5T985|Q5T985_HUMAN	85.28	4	3	3

499	sp|P19823|ITIH2_HUMAN	85.28	4	3	3
500	sp|O43493-2|TGON2_HUMAN	84.41	10	3	3
501	sp|O43493-5|TGON2_HUMAN	84.41	10	3	3
502	tr|J3KQ45|J3KQ45_HUMAN	84.41	10	3	3
503	sp|O43493-3|TGON2_HUMAN	84.41	10	3	3
504	sp|O43493|TGON2_HUMAN	84.41	9	3	3
505	sp|Q8NCW5|NNRE_HUMAN	84.12	10	3	3
506	sp|Q9Y3E1|HDGR3_HUMAN	83.97	16	3	3
507	sp|Q9Y5Z4-2|HEBP2_HUMAN	83.82	16	3	3
508	sp|Q9Y5Z4|HEBP2_HUMAN	83.82	14	3	3
509	tr|H3BVI7|H3BVI7_HUMAN	83.78	5	3	3
510	tr|H3BNC6|H3BNC6_HUMAN	83.78	4	3	3
511	tr|Q9UII8|Q9UII8_HUMAN	83.78	3	3	3
512	sp|P12830|CADH1_HUMAN	83.78	2	3	3
513	sp|P51858|HDGF_HUMAN	83.32	15	3	3
514	tr|H3BM42|H3BM42_HUMAN	83.16	4	3	3
515	sp|Q92896|GSLG1_HUMAN	83.16	3	3	3
516	sp|Q92896-3|GSLG1_HUMAN	83.16	3	3	3
517	sp|Q92896-2|GSLG1_HUMAN	83.16	2	3	3
518	sp|Q01459|DIAC_HUMAN	83.10	14	4	4
519	tr|E9PHT9|E9PHT9_HUMAN	82.96	18	3	3
520	sp|P08758|ANXA5_HUMAN	82.96	9	3	3
521	sp|P04083|ANXA1_HUMAN	82.63	9	3	3
522	sp|P00492|HPRT_HUMAN	82.31	16	3	3
523	sp|P30101|PDIA3_HUMAN	80.50	6	3	3
524	tr|B4DS13|B4DS13_HUMAN	80.19	7	3	3
525	sp|P23588|IF4B_HUMAN	80.19	7	3	3
526	tr|E7EX17|E7EX17_HUMAN	80.19	7	3	3
527	tr|H0YN49|H0YN49_HUMAN	80.03	21	3	3
528	tr|H0YMZ3|H0YMZ3_HUMAN	80.03	11	3	3
529	sp|Q9NPR2-2|SEM4B_HUMAN	80.03	7	3	3
530	sp|Q9NPR2|SEM4B_HUMAN	80.03	6	3	3
531	tr|J3KNP4|J3KNP4_HUMAN	80.03	6	3	3
532	sp|Q9C0C2-2|TB182_HUMAN	79.63	7	4	4
533	sp|Q9C0C2|TB182_HUMAN	79.63	3	4	4
534	sp|P01023|A2MG_HUMAN	79.57	3	4	4
535	sp|Q9UHD9|UBQL2_HUMAN	79.22	5	3	3
536	sp|Q9UMX0-2|UBQL1_HUMAN	79.22	6	3	3
537	sp|Q9UMX0|UBQL1_HUMAN	79.22	6	3	3
538	tr|Q6ZR19|Q6ZR19_HUMAN	79.05	3	4	4
539	sp|P23467-4|PTPRB_HUMAN	79.05	2	4	4
540	tr|F8VSD5|F8VSD5_HUMAN	79.05	3	4	4
541	sp|P23467|PTPRB_HUMAN	79.05	2	4	4
542	sp|P23467-3|PTPRB_HUMAN	79.05	2	4	4
543	tr|J3QT52|J3QT52_HUMAN	79.05	2	4	4
544	sp|P23467-2|PTPRB_HUMAN	79.05	2	4	4
545	sp|P15291-2|B4GT1_HUMAN	78.58	8	3	3
546	sp|P15291|B4GT1_HUMAN	78.58	8	3	3
547	tr|D6RDU5|D6RDU5_HUMAN	78.29	18	5	4
548	tr|D6RGI3|D6RGI3_HUMAN	78.29	16	5	4

549	sp|Q9NVA2|SEP11_HUMAN	78.29	16	5	4
550	tr|D6RER5|D6RER5_HUMAN	78.29	16	5	4
551	sp|Q9NVA2-2|SEP11_HUMAN	78.29	15	5	4
552	sp|O94907|DKK1_HUMAN	78.28	9	3	3
553	sp|Q13442|HAP28_HUMAN	77.87	22	3	3
554	sp|Q14696|MESD_HUMAN	77.66	18	3	3
555	sp|O75874|IDHC_HUMAN	76.62	11	4	4
556	sp|Q9UHL4|DPP2_HUMAN	76.41	7	3	3
557	sp|P98172|EFNB1_HUMAN	76.38	9	4	4
558	sp|Q9NZU0|FLRT3_HUMAN	75.94	8	4	4
559	tr|E9PP14|E9PP14_HUMAN	75.30	15	3	3
560	tr|E9PKL9|E9PKL9_HUMAN	75.30	11	3	3
561	sp|Q13630|FCL_HUMAN	75.30	9	3	3
562	sp|Q8IV08|PLD3_HUMAN	74.09	6	3	3
563	sp|Q15046|SYK_HUMAN	73.92	7	3	3
564	sp|Q15046-2|SYK_HUMAN	69.86	6	3	3
565	sp|P08582|TRFM_HUMAN	73.18	5	4	3
566	sp|Q13938|CAYP1_HUMAN	72.84	17	3	3
567	tr|K7EL21|K7EL21_HUMAN	72.84	12	3	3
568	sp|O00764-2|PDXK_HUMAN	72.75	13	3	3
569	sp|O00764|PDXK_HUMAN	72.75	12	3	3
570	tr|C9JMM0|C9JMM0_HUMAN	71.36	32	3	3
571	tr|B8ZZ43|B8ZZ43_HUMAN	71.36	20	3	3
572	tr|S4R2Y4|S4R2Y4_HUMAN	71.36	19	3	3
573	sp|Q13185|CBX3_HUMAN	71.36	11	3	3
574	sp|P81605|DCD_HUMAN	71.18	28	3	3
575	sp|P81605-2|DCD_HUMAN	71.18	26	3	3
576	sp|P02750|A2GL_HUMAN	71.13	7	3	3
577	sp|P29622|KAIN_HUMAN	68.50	6	3	3
578	tr|H7BZT7|H7BZT7_HUMAN	67.58	19	3	3
579	tr|X6RA14|X6RA14_HUMAN	67.58	17	3	3
580	sp|P10768|ESTD_HUMAN	67.58	15	3	3
581	sp|P15529-15|MCP_HUMAN	59.08	5	3	3
582	sp|P15529-9|MCP_HUMAN	59.08	5	3	3
583	sp|P15529-7|MCP_HUMAN	59.08	5	3	3
584	sp|P15529-12|MCP_HUMAN	59.08	5	3	3
585	sp|P15529-14|MCP_HUMAN	59.08	5	3	3
586	sp|P15529-8|MCP_HUMAN	59.08	5	3	3
587	sp|P15529-4|MCP_HUMAN	59.08	5	3	3
588	sp|P15529-6|MCP_HUMAN	59.08	5	3	3
589	sp|P15529-11|MCP_HUMAN	59.08	5	3	3
590	sp|P15529-13|MCP_HUMAN	59.08	4	3	3
591	sp|P15529-3|MCP_HUMAN	59.08	4	3	3
592	sp|P15529-5|MCP_HUMAN	59.08	4	3	3
593	sp|P15529-10|MCP_HUMAN	59.08	4	3	3
594	sp|P15529|MCP_HUMAN	59.08	4	3	3
595	sp|P15529-2|MCP_HUMAN	59.08	4	3	3

spectrometry

Description

Filamin-A OS=Homo sapiens GN=FLNA PE=1 SV=4
Isoform 2 of Filamin-A OS=Homo sapiens GN=FLNA
Filamin-A OS=Homo sapiens GN=FLNA PE=1 SV=1
Complement C3 OS=Homo sapiens GN=C3 PE=1 SV=2
Isoform 4 of Plectin OS=Homo sapiens GN=PLEC
Keratin type I cytoskeletal 9 OS=Homo sapiens GN=KRT9 PE=1 SV=3
Pyro-glu from Q
Isoform 8 of Filamin-B OS=Homo sapiens GN=FLNB
Filamin-B OS=Homo sapiens GN=FLNB PE=1 SV=2
Isoform 9 of Filamin-B OS=Homo sapiens GN=FLNB
Isoform 2 of Filamin-B OS=Homo sapiens GN=FLNB
Pyro-glu from Q
Isoform 6 of Calpastatin OS=Homo sapiens GN=CAST
Isoform 7 of Calpastatin OS=Homo sapiens GN=CAST
Calpastatin OS=Homo sapiens GN=CAST PE=1 SV=1
Calpastatin OS=Homo sapiens GN=CAST PE=1 SV=1
Isoform 9 of Calpastatin OS=Homo sapiens GN=CAST
Isoform 2 of Keratin type II cytoskeletal 8 OS=Homo sapiens GN=KRT8
Keratin type II cytoskeletal 8 OS=Homo sapiens GN=KRT8 PE=1 SV=7
Heat shock 70 kDa protein 1A/1B OS=Homo sapiens GN=HSPA1A PE=1 SV=5
Isoform 2 of Heat shock 70 kDa protein 1A/1B OS=Homo sapiens GN=HSPA1A
Ezrin OS=Homo sapiens GN=EZR PE=1 SV=4
Heat shock cognate 71 kDa protein OS=Homo sapiens GN=HSPA8 PE=1 SV=1
Semaphorin-7A OS=Homo sapiens GN=SEMA7A PE=1 SV=1
Laminin subunit gamma-2 OS=Homo sapiens GN=LAMC2 PE=1 SV=2
Olfactomedin-4 OS=Homo sapiens GN=OLFM4 PE=1 SV=1
Osteopontin OS=Homo sapiens GN=SPP1 PE=1 SV=1
Isoform 5 of Osteopontin OS=Homo sapiens GN=SPP1
Isoform B of Osteopontin OS=Homo sapiens GN=SPP1
Isoform D of Osteopontin OS=Homo sapiens GN=SPP1
Isoform C of Osteopontin OS=Homo sapiens GN=SPP1
Laminin subunit beta-3 OS=Homo sapiens GN=LAMB3 PE=1 SV=1
Keratin type II cytoskeletal 1 OS=Homo sapiens GN=KRT1 PE=1 SV=6
Isoform 4 of Gelsolin OS=Homo sapiens GN=GSN
Isoform 2 of Gelsolin OS=Homo sapiens GN=GSN
Gelsolin OS=Homo sapiens GN=GSN PE=1 SV=1
Isoform 3 of Gelsolin OS=Homo sapiens GN=GSN
Lipolysis-stimulated lipoprotein receptor (Fragment) OS=Homo sapiens GN=LSR PE=1 SV=1
Lipolysis-stimulated lipoprotein receptor OS=Homo sapiens GN=LSR PE=1 SV=1
Isoform 5 of Lipolysis-stimulated lipoprotein receptor OS=Homo sapiens GN=LSR
Lipolysis-stimulated lipoprotein receptor OS=Homo sapiens GN=LSR PE=1 SV=4
Isoform 4 of Lipolysis-stimulated lipoprotein receptor OS=Homo sapiens GN=LSR
Keratin type I cytoskeletal 19 OS=Homo sapiens GN=KRT19 PE=1 SV=4
CD166 antigen OS=Homo sapiens GN=ALCAM PE=1 SV=2
Isoform 2 of CD166 antigen OS=Homo sapiens GN=ALCAM
Keratin type II cytoskeletal 2 epidermal OS=Homo sapiens GN=KRT2 PE=1 SV=2
Complement factor B OS=Homo sapiens GN=CFB PE=1 SV=2
Complement factor B OS=Homo sapiens GN=CFB PE=2 SV=1

Keratin type I cytoskeletal 18 OS=Homo sapiens GN=KRT18 PE=1 SV=2
Laminin subunit alpha-3 OS=Homo sapiens GN=LAMA3 PE=1 SV=2
Sulfhydryl oxidase 1 OS=Homo sapiens GN=QSOX1 PE=1 SV=3
Protein CDV3 homolog OS=Homo sapiens GN=CDV3 PE=1 SV=1
Cation-independent mannose-6-phosphate receptor OS=Homo sapiens GN=IGF2R PE=1 SV=3
L-lactate dehydrogenase B chain OS=Homo sapiens GN=LDHB PE=1 SV=2
Actin cytoplasmic 1 OS=Homo sapiens GN=ACTB PE=1 SV=1
Actin cytoplasmic 2 OS=Homo sapiens GN=ACTG1 PE=1 SV=1
Pyruvate kinase PKM OS=Homo sapiens GN=PKM PE=1 SV=4
Keratin type I cytoskeletal 10 OS=Homo sapiens GN=KRT10 PE=1 SV=6
Microtubule-associated protein 4 OS=Homo sapiens GN=MAP4 PE=1 SV=3
Microtubule-associated protein OS=Homo sapiens GN=MAP4 PE=1 SV=1
Neuroblast differentiation-associated protein AHNAK OS=Homo sapiens GN=AHNAK PE=1 SV=2
Isoform 2 of Fructose-bisphosphate aldolase A OS=Homo sapiens GN=ALDOA
Fructose-bisphosphate aldolase OS=Homo sapiens GN=ALDOA PE=1 SV=1
Fructose-bisphosphate aldolase A OS=Homo sapiens GN=ALDOA PE=1 SV=2
Isoform 6 of Peptidyl-glycine alpha-amidating monooxygenase OS=Homo sapiens GN=PAM
Isoform 5 of Peptidyl-glycine alpha-amidating monooxygenase OS=Homo sapiens GN=PAM
Peptidyl-glycine alpha-amidating monooxygenase OS=Homo sapiens GN=PAM PE=1 SV=2
Isoform 2 of Peptidyl-glycine alpha-amidating monooxygenase OS=Homo sapiens GN=PAM
Dystroglycan OS=Homo sapiens GN=DAG1 PE=1 SV=2
Receptor-type tyrosine-protein phosphatase F OS=Homo sapiens GN=PTPRF PE=1 SV=2
Isoform 2 of Receptor-type tyrosine-protein phosphatase F OS=Homo sapiens GN=PTPRF
Isoform 2 of Procollagen-lysine 2-oxoglutarate 5-dioxygenase 2 OS=Homo sapiens GN=PLOD2
Procollagen-lysine 2-oxoglutarate 5-dioxygenase 2 OS=Homo sapiens GN=PLOD2 PE=1 SV=2
Vinculin OS=Homo sapiens GN=VCL PE=1 SV=4
Isoform 1 of Vinculin OS=Homo sapiens GN=VCL
Intercellular adhesion molecule 1 OS=Homo sapiens GN=ICAM1 PE=1 SV=2
Collagen alpha-1(VI) chain OS=Homo sapiens GN=COL6A1 PE=1 SV=3
Isoform 5 of Inactive tyrosine-protein kinase 7 OS=Homo sapiens GN=PTK7
Isoform 2 of Inactive tyrosine-protein kinase 7 OS=Homo sapiens GN=PTK7
Isoform 6 of Inactive tyrosine-protein kinase 7 OS=Homo sapiens GN=PTK7
Inactive tyrosine-protein kinase 7 OS=Homo sapiens GN=PTK7 PE=1 SV=2
Keratin type II cytoskeletal 7 OS=Homo sapiens GN=KRT7 PE=1 SV=5
Alpha-enolase OS=Homo sapiens GN=ENO1 PE=1 SV=2
Src substrate cortactin OS=Homo sapiens GN=CTTN PE=1 SV=2
Lamin B2 isoform CRA_a OS=Homo sapiens GN=LMNB2 PE=1 SV=1
Lamin-B2 OS=Homo sapiens GN=LMNB2 PE=1 SV=3
Vesicular integral-membrane protein VIP36 OS=Homo sapiens GN=LMAN2 PE=1 SV=1
Vesicular integral-membrane protein VIP36 OS=Homo sapiens GN=LMAN2 PE=1 SV=1
Moesin OS=Homo sapiens GN=MSN PE=1 SV=3
Dipeptidyl peptidase 4 OS=Homo sapiens GN=DPP4 PE=1 SV=2
Hematological and neurological expressed 1 protein OS=Homo sapiens GN=HN1 PE=1 SV=3
Elongation factor 2 OS=Homo sapiens GN=EEF2 PE=1 SV=4
Complement factor H OS=Homo sapiens GN=CFH PE=1 SV=4
Isoform 3 of Agrin OS=Homo sapiens GN=AGRN
Isoform 6 of Agrin OS=Homo sapiens GN=AGRN
Hepatocyte growth factor receptor OS=Homo sapiens GN=MET PE=1 SV=4
Keratin type I cytoskeletal 14 OS=Homo sapiens GN=KRT14 PE=1 SV=4
Receptor tyrosine-protein kinase erbB-2 OS=Homo sapiens GN=ERBB2 PE=1 SV=1

Kinectin OS=Homo sapiens GN=KTN1 PE=1 SV=1
Isoform 3 of Kinectin OS=Homo sapiens GN=KTN1
Isoform 4 of Kinectin OS=Homo sapiens GN=KTN1
Isoform 2 of Kinectin OS=Homo sapiens GN=KTN1
Gamma-glutamylcyclotransferase OS=Homo sapiens GN=GGCT PE=1 SV=1
Peroxiredoxin-1 OS=Homo sapiens GN=PRDX1 PE=1 SV=1
Nicotinamide phosphoribosyltransferase OS=Homo sapiens GN=NAMPT PE=1 SV=1
Isoform 2 of Cytosol aminopeptidase OS=Homo sapiens GN=LAP3
Cytosol aminopeptidase OS=Homo sapiens GN=LAP3 PE=1 SV=3
Procollagen-lysine 2-oxoglutarate 5-dioxygenase 1 OS=Homo sapiens GN=PLOD1 PE=1 SV=2
CD109 antigen OS=Homo sapiens GN=CD109 PE=1 SV=2
Isoform 4 of CD109 antigen OS=Homo sapiens GN=CD109
Phosphoglycerate mutase 1 OS=Homo sapiens GN=PGAM1 PE=1 SV=2
Solute carrier family 12 member 2 OS=Homo sapiens GN=SLC12A2 PE=1 SV=1
Isoform 2 of Solute carrier family 12 member 2 OS=Homo sapiens GN=SLC12A2
Solute carrier family 12 (Sodium/potassium/chloride transporters) member 2 isoform CRA_a OS=Homo sapie
Isoform 3 of Neural cell adhesion molecule L1 OS=Homo sapiens GN=L1CAM
Neural cell adhesion molecule L1 OS=Homo sapiens GN=L1CAM PE=1 SV=1
Neural cell adhesion molecule L1 OS=Homo sapiens GN=L1CAM PE=1 SV=2
Neural cell adhesion molecule L1 OS=Homo sapiens GN=L1CAM PE=1 SV=1
Isoform 2 of Neural cell adhesion molecule L1 OS=Homo sapiens GN=L1CAM
Isoform 2 of Xaa-Pro dipeptidase OS=Homo sapiens GN=PEPD
Xaa-Pro dipeptidase OS=Homo sapiens GN=PEPD PE=1 SV=3
Plasminogen activator inhibitor 2 OS=Homo sapiens GN=SERPINB2 PE=1 SV=2
Transcobalamin-1 OS=Homo sapiens GN=TCN1 PE=1 SV=2
Heterogeneous nuclear ribonucleoprotein L OS=Homo sapiens GN=HNRNPL PE=1 SV=2
Multiple epidermal growth factor-like domains protein 8 OS=Homo sapiens GN=MEGF8 PE=1 SV=2
Isoform 2 of Rab GDP dissociation inhibitor beta OS=Homo sapiens GN=GDI2
Rab GDP dissociation inhibitor beta OS=Homo sapiens GN=GDI2 PE=1 SV=1
Rab GDP dissociation inhibitor beta OS=Homo sapiens GN=GDI2 PE=1 SV=2
Activator of 90 kDa heat shock protein ATPase homolog 1 OS=Homo sapiens GN=AHSA1 PE=1 SV=1
Laminin subunit gamma-1 OS=Homo sapiens GN=LAMC1 PE=1 SV=3
Leukocyte elastase inhibitor OS=Homo sapiens GN=SERPINB1 PE=1 SV=1
Cytosolic non-specific dipeptidase OS=Homo sapiens GN=CNDP2 PE=1 SV=2
Na(+)/H(+) exchange regulatory cofactor NHE-RF1 OS=Homo sapiens GN=SLC9A3R1 PE=1 SV=4
Plexin-B2 OS=Homo sapiens GN=PLXNB2 PE=1 SV=3
Calcyclin-binding protein OS=Homo sapiens GN=CACYBP PE=1 SV=2
Basement membrane-specific heparan sulfate proteoglycan core protein OS=Homo sapiens GN=HSPG2 PE=1 S
Nucleolin OS=Homo sapiens GN=NCL PE=1 SV=3
Lamin-B1 OS=Homo sapiens GN=LMNB1 PE=1 SV=1
Lamin-B1 OS=Homo sapiens GN=LMNB1 PE=1 SV=2
Platelet-derived growth factor D OS=Homo sapiens GN=PDGFD PE=1 SV=1
Isoform 6 of Thioredoxin reductase 1 cytoplasmic OS=Homo sapiens GN=TXNRD1
Thioredoxin reductase 1 cytoplasmic OS=Homo sapiens GN=TXNRD1 PE=1 SV=1
Thioredoxin reductase 1 cytoplasmic OS=Homo sapiens GN=TXNRD1 PE=1 SV=1
Thioredoxin reductase 1 cytoplasmic OS=Homo sapiens GN=TXNRD1 PE=1 SV=1
Isoform 5 of Thioredoxin reductase 1 cytoplasmic OS=Homo sapiens GN=TXNRD1
Thioredoxin reductase 1 cytoplasmic OS=Homo sapiens GN=TXNRD1 PE=1 SV=1
Isoform 2 of Thioredoxin reductase 1 cytoplasmic OS=Homo sapiens GN=TXNRD1
Thioredoxin reductase 1 cytoplasmic OS=Homo sapiens GN=TXNRD1 PE=1 SV=1

Isoform 4 of Thioredoxin reductase 1 cytoplasmic OS=Homo sapiens GN=TXNRD1
Thioredoxin reductase 1 cytoplasmic OS=Homo sapiens GN=TXNRD1 PE=1 SV=1
Thioredoxin reductase 1 cytoplasmic OS=Homo sapiens GN=TXNRD1 PE=1 SV=2
Isoform 3 of Thioredoxin reductase 1 cytoplasmic OS=Homo sapiens GN=TXNRD1
Thioredoxin reductase 1 cytoplasmic OS=Homo sapiens GN=TXNRD1 PE=1 SV=3
Dihydropyrimidine dehydrogenase [NADP(+)] OS=Homo sapiens GN=DPYD PE=1 SV=2
Nectin-4 OS=Homo sapiens GN=PVRL4 PE=1 SV=1
Stress-70 protein mitochondrial OS=Homo sapiens GN=HSPA9 PE=1 SV=2
Isoform 3 of Heterogeneous nuclear ribonucleoprotein K OS=Homo sapiens GN=HNRNPK
Isoform 2 of Heterogeneous nuclear ribonucleoprotein K OS=Homo sapiens GN=HNRNPK
Heterogeneous nuclear ribonucleoprotein K OS=Homo sapiens GN=HNRNPK PE=1 SV=1
High mobility group protein HMG-I/HMG-Y OS=Homo sapiens GN=HMGA1 PE=1 SV=3
Heterogeneous nuclear ribonucleoproteins A2/B1 OS=Homo sapiens GN=HNRNPA2B1 PE=1 SV=2
Protein RCC2 OS=Homo sapiens GN=RCC2 PE=1 SV=2
Desmoglein-2 OS=Homo sapiens GN=DSG2 PE=1 SV=2
Leukotriene A-4 hydrolase OS=Homo sapiens GN=LTA4H PE=1 SV=2
Isoform 5 of Clusterin OS=Homo sapiens GN=CLU
Clusterin OS=Homo sapiens GN=CLU PE=1 SV=1
Isoform 2 of Clusterin OS=Homo sapiens GN=CLU
ADAM9 protein OS=Homo sapiens GN=ADAM9 PE=2 SV=1
Disintegrin and metalloproteinase domain-containing protein 9 OS=Homo sapiens GN=ADAM9 PE=3 SV=1
Isoform 2 of Disintegrin and metalloproteinase domain-containing protein 9 OS=Homo sapiens GN=ADAM9
Disintegrin and metalloproteinase domain-containing protein 9 OS=Homo sapiens GN=ADAM9 PE=1 SV=1
Insulin-like growth factor-binding protein 3 OS=Homo sapiens GN=IGFBP3 PE=1 SV=2
Isoform 2 of Insulin-like growth factor-binding protein 3 OS=Homo sapiens GN=IGFBP3
78 kDa glucose-regulated protein OS=Homo sapiens GN=HSPA5 PE=1 SV=2
Isoform 2 of Basigin OS=Homo sapiens GN=BSG
Transforming growth factor-beta-induced protein ig-h3 OS=Homo sapiens GN=TGFBI PE=1 SV=1
Transforming growth factor-beta-induced protein ig-h3 OS=Homo sapiens GN=TGFBI PE=4 SV=1
Isoform 2 of Epithelial discoidin domain-containing receptor 1 OS=Homo sapiens GN=DDR1
Isoform 5 of Epithelial discoidin domain-containing receptor 1 OS=Homo sapiens GN=DDR1
Isoform 4 of Epithelial discoidin domain-containing receptor 1 OS=Homo sapiens GN=DDR1
Epithelial discoidin domain-containing receptor 1 OS=Homo sapiens GN=DDR1 PE=1 SV=1
Cathepsin D OS=Homo sapiens GN=CTSD PE=1 SV=1
Transketolase OS=Homo sapiens GN=TKT PE=1 SV=1
Transketolase OS=Homo sapiens GN=TKT PE=1 SV=3
Isoform 2 of Transketolase OS=Homo sapiens GN=TKT
Keratin type II cytoskeletal 5 OS=Homo sapiens GN=KRT5 PE=1 SV=3
Rho GDP-dissociation inhibitor 1 (Fragment) OS=Homo sapiens GN=ARHGDIA PE=1 SV=2
Rho GDP-dissociation inhibitor 1 OS=Homo sapiens GN=ARHGDIA PE=1 SV=3
Rho GDP-dissociation inhibitor 1 OS=Homo sapiens GN=ARHGDIA PE=1 SV=1
Spectrin beta chain non-erythrocytic 1 OS=Homo sapiens GN=SPTBN1 PE=1 SV=2
Stathmin OS=Homo sapiens GN=STMN1 PE=1 SV=3
Isoform 2 of Stathmin OS=Homo sapiens GN=STMN1
Isoform 3 of Soluble calcium-activated nucleotidase 1 OS=Homo sapiens GN=CANT1
Soluble calcium-activated nucleotidase 1 OS=Homo sapiens GN=CANT1 PE=1 SV=1
UV excision repair protein RAD23 homolog B OS=Homo sapiens GN=RAD23B PE=1 SV=1
Isoform 2 of Attractin OS=Homo sapiens GN=ATRN
Attractin OS=Homo sapiens GN=ATRN PE=1 SV=2
Isoform 2 of Glutathione synthetase OS=Homo sapiens GN=GSS

Glutathione synthetase OS=Homo sapiens GN=GSS PE=1 SV=1
Urokinase-type plasminogen activator chain B OS=Homo sapiens GN=PLAU PE=3 SV=2
Thimet oligopeptidase OS=Homo sapiens GN=THOP1 PE=1 SV=2
ERO1-like protein alpha OS=Homo sapiens GN=ERO1L PE=1 SV=2
Vacuolar protein sorting-associated protein 26A OS=Homo sapiens GN=VPS26A PE=1 SV=2
Isoform 2 of Nectin-3 OS=Homo sapiens GN=PVRL3
Isoform 3 of Nectin-3 OS=Homo sapiens GN=PVRL3
Nectin-3 OS=Homo sapiens GN=PVRL3 PE=1 SV=1
Isoform 3 of Heterogeneous nuclear ribonucleoprotein D0 OS=Homo sapiens GN=HNRNPD
Heterogeneous nuclear ribonucleoprotein D0 OS=Homo sapiens GN=HNRNPD PE=1 SV=1
HLA class II histocompatibility antigen DR alpha chain OS=Homo sapiens GN=HLA-DRA PE=1 SV=1
HLA class II histocompatibility antigen DR alpha chain OS=Homo sapiens GN=HLA-DRA PE=1 SV=1
Lamina-associated polypeptide 2 isoforms beta/gamma OS=Homo sapiens GN=TMPO PE=1 SV=2
Isoform 2 of Transcription intermediary factor 1-beta OS=Homo sapiens GN=TRIM28
Transcription intermediary factor 1-beta OS=Homo sapiens GN=TRIM28 PE=1 SV=5
Ephrin type-B receptor 2 OS=Homo sapiens GN=EPHB2 PE=1 SV=1
Isoform 2 of Ephrin type-B receptor 2 OS=Homo sapiens GN=EPHB2
Isoform 3 of Ephrin type-B receptor 2 OS=Homo sapiens GN=EPHB2
Ephrin type-B receptor 2 OS=Homo sapiens GN=EPHB2 PE=1 SV=5
Stress-induced-phosphoprotein 1 OS=Homo sapiens GN=STIP1 PE=1 SV=1
Lysosomal alpha-mannosidase OS=Homo sapiens GN=MAN2B1 PE=1 SV=3
Isoform 2 of Lysosomal alpha-mannosidase OS=Homo sapiens GN=MAN2B1
Capping protein (Actin filament) muscle Z-line beta isoform CRA_a OS=Homo sapiens GN=CAPZB PE=1 SV=1
Isoform 2 of F-actin-capping protein subunit beta OS=Homo sapiens GN=CAPZB
Capping protein (Actin filament) muscle Z-line beta isoform CRA_d OS=Homo sapiens GN=CAPZB PE=1 SV=1
HLA class II histocompatibility antigen DRB1-4 beta chain OS=Homo sapiens GN=HLA-DRB1 PE=1 SV=1
Phosphoglycerate kinase 1 OS=Homo sapiens GN=PGK1 PE=1 SV=3
Isoform Short of TATA-binding protein-associated factor 2N OS=Homo sapiens GN=TAF15
TATA-binding protein-associated factor 2N OS=Homo sapiens GN=TAF15 PE=1 SV=1
Elongation factor 1-alpha 2 OS=Homo sapiens GN=EEF1A2 PE=1 SV=1
Elongation factor 1-alpha 1 OS=Homo sapiens GN=EEF1A1 PE=1 SV=1
Putative elongation factor 1-alpha-like 3 OS=Homo sapiens GN=EEF1A1P5 PE=5 SV=1
10 kDa heat shock protein mitochondrial OS=Homo sapiens GN=HSPE1 PE=1 SV=2
Cell adhesion molecule 4 OS=Homo sapiens GN=CADM4 PE=1 SV=1
Lysosomal alpha-glucosidase OS=Homo sapiens GN=GAA PE=1 SV=4
Isoform 3 of Apoptosis-inducing factor 1 mitochondrial OS=Homo sapiens GN=AIFM1
Apoptosis-inducing factor 1 mitochondrial OS=Homo sapiens GN=AIFM1 PE=1 SV=1
Retinal dehydrogenase 2 OS=Homo sapiens GN=ALDH1A2 PE=1 SV=3
Peptidyl-prolyl cis-trans isomerase A OS=Homo sapiens GN=PPIA PE=1 SV=2
Glucosamine (N-acetyl)-6-sulfatase (Sanfilippo disease IIID) isoform CRA_b OS=Homo sapiens GN=GNS PE=1 S
N-acetylglucosamine-6-sulfatase OS=Homo sapiens GN=GNS PE=1 SV=1
N-acetylglucosamine-6-sulfatase OS=Homo sapiens GN=GNS PE=1 SV=3
N-acetylglucosamine-6-sulfatase OS=Homo sapiens GN=GNS PE=1 SV=1
PEST proteolytic signal-containing nuclear protein OS=Homo sapiens GN=PCNP PE=1 SV=2
Keratin type I cytoskeletal 16 OS=Homo sapiens GN=KRT16 PE=1 SV=4
Dihydrolipoyl dehydrogenase mitochondrial OS=Homo sapiens GN=DLD PE=1 SV=1
Dihydrolipoyl dehydrogenase mitochondrial OS=Homo sapiens GN=DLD PE=1 SV=1
Dihydrolipoyl dehydrogenase mitochondrial OS=Homo sapiens GN=DLD PE=1 SV=2
6-phosphogluconate dehydrogenase decarboxylating (Fragment) OS=Homo sapiens GN=PGD PE=1 SV=1
6-phosphogluconate dehydrogenase decarboxylating (Fragment) OS=Homo sapiens GN=PGD PE=1 SV=1

6-phosphogluconate dehydrogenase decarboxylating (Fragment) OS=Homo sapiens GN=PGD PE=1 SV=1
Uncharacterized protein OS=Homo sapiens GN=PGD PE=1 SV=1
6-phosphogluconate dehydrogenase decarboxylating OS=Homo sapiens GN=PGD PE=1 SV=1
6-phosphogluconate dehydrogenase decarboxylating OS=Homo sapiens GN=PGD PE=1 SV=3
Alpha-mannosidase 2 OS=Homo sapiens GN=MAN2A1 PE=1 SV=2
Alpha-2-HS-glycoprotein OS=Homo sapiens GN=AHSG PE=1 SV=1
Cathepsin Z OS=Homo sapiens GN=CTSZ PE=1 SV=1
Cell surface glycoprotein MUC18 OS=Homo sapiens GN=MCAM PE=1 SV=2
Isoform 6 of Roundabout homolog 1 OS=Homo sapiens GN=ROBO1
Isoform 5 of Roundabout homolog 1 OS=Homo sapiens GN=ROBO1
Isoform 4 of Roundabout homolog 1 OS=Homo sapiens GN=ROBO1
Isoform 3 of Roundabout homolog 1 OS=Homo sapiens GN=ROBO1
Roundabout homolog 1 OS=Homo sapiens GN=ROBO1 PE=1 SV=1
Isoform 2 of Roundabout homolog 1 OS=Homo sapiens GN=ROBO1
Heterogeneous nuclear ribonucleoprotein A/B OS=Homo sapiens GN=HNRNPAB PE=1 SV=1
Isoform 3 of Heterogeneous nuclear ribonucleoprotein A/B OS=Homo sapiens GN=HNRNPAB
Heterogeneous nuclear ribonucleoprotein A/B OS=Homo sapiens GN=HNRNPAB PE=1 SV=1
Heterogeneous nuclear ribonucleoprotein A/B OS=Homo sapiens GN=HNRNPAB PE=1 SV=1
Isoform 2 of Heterogeneous nuclear ribonucleoprotein A/B OS=Homo sapiens GN=HNRNPAB
Isoform 2 of Adenylyl cyclase-associated protein 1 OS=Homo sapiens GN=CAP1
Adenylyl cyclase-associated protein 1 OS=Homo sapiens GN=CAP1 PE=1 SV=5
WD repeat-containing protein 1 OS=Homo sapiens GN=WDR1 PE=1 SV=4
Alpha-N-acetylglucosaminidase OS=Homo sapiens GN=NAGLU PE=1 SV=2
Glyceraldehyde-3-phosphate dehydrogenase OS=Homo sapiens GN=GAPDH PE=1 SV=3
Isoform 2 of Heterogeneous nuclear ribonucleoprotein A1 OS=Homo sapiens GN=HNRNPA1
Heterogeneous nuclear ribonucleoprotein A1 OS=Homo sapiens GN=HNRNPA1 PE=1 SV=5
Heterogeneous nuclear ribonucleoprotein A1 OS=Homo sapiens GN=HNRNPA1 PE=1 SV=2
Isoform A1-A of Heterogeneous nuclear ribonucleoprotein A1 OS=Homo sapiens GN=HNRNPA1
Isoform Gamma of Poliovirus receptor OS=Homo sapiens GN=PVR
Isoform Beta of Poliovirus receptor OS=Homo sapiens GN=PVR
Isoform Delta of Poliovirus receptor OS=Homo sapiens GN=PVR
Poliovirus receptor OS=Homo sapiens GN=PVR PE=1 SV=2
Keratin type I cytoskeletal 13 OS=Homo sapiens GN=KRT13 PE=1 SV=4
Splicing factor 3B subunit 2 OS=Homo sapiens GN=SF3B2 PE=1 SV=1
Splicing factor 3B subunit 2 OS=Homo sapiens GN=SF3B2 PE=1 SV=2
Isoform 3 of Tubulointerstitial nephritis antigen-like OS=Homo sapiens GN=TINAGL1
Tubulointerstitial nephritis antigen-like OS=Homo sapiens GN=TINAGL1 PE=1 SV=1
Thioredoxin domain-containing protein 17 OS=Homo sapiens GN=TXNDC17 PE=1 SV=1
Isoform 4 of Heterogeneous nuclear ribonucleoprotein Q OS=Homo sapiens GN=SYNCRIP
Isoform 3 of Heterogeneous nuclear ribonucleoprotein Q OS=Homo sapiens GN=SYNCRIP
Isoform 2 of Heterogeneous nuclear ribonucleoprotein Q OS=Homo sapiens GN=SYNCRIP
Heterogeneous nuclear ribonucleoprotein Q OS=Homo sapiens GN=SYNCRIP PE=1 SV=2
Periplakin (Fragment) OS=Homo sapiens GN=PPL PE=1 SV=1
Periplakin OS=Homo sapiens GN=PPL PE=1 SV=1
Periplakin OS=Homo sapiens GN=PPL PE=1 SV=4
Annexin A3 OS=Homo sapiens GN=ANXA3 PE=1 SV=3
Coatomer protein complex subunit beta 2 (Beta prime) isoform CRA_b OS=Homo sapiens GN=COPB2 PE=1 S\
Coatomer subunit beta' OS=Homo sapiens GN=COPB2 PE=1 SV=2
Heterogeneous nuclear ribonucleoprotein D-like OS=Homo sapiens GN=HNRNPDL PE=1 SV=3
Isoform 3 of Heterogeneous nuclear ribonucleoprotein D-like OS=Homo sapiens GN=HNRNPDL

Isoform 2 of Heterogeneous nuclear ribonucleoprotein D-like OS=Homo sapiens GN=HNRNPDL
Serum albumin OS=Homo sapiens GN=ALB PE=1 SV=2
Eukaryotic translation initiation factor 4E-binding protein 1 OS=Homo sapiens GN=EIF4EBP1 PE=1 SV=3
N(G) N(G)-dimethylarginine dimethylaminohydrolase 1 OS=Homo sapiens GN=DDAH1 PE=1 SV=3
Isoform 2 of Carcinoembryonic antigen-related cell adhesion molecule 1 OS=Homo sapiens GN=CEACAM1
Isoform 8 of Carcinoembryonic antigen-related cell adhesion molecule 1 OS=Homo sapiens GN=CEACAM1
Isoform 10 of Carcinoembryonic antigen-related cell adhesion molecule 1 OS=Homo sapiens GN=CEACAM1
Carcinoembryonic antigen-related cell adhesion molecule 1 OS=Homo sapiens GN=CEACAM1 PE=1 SV=2
Acid ceramidase OS=Homo sapiens GN=ASAH1 PE=1 SV=1
Acid ceramidase OS=Homo sapiens GN=ASAH1 PE=1 SV=5
Isoform 2 of Acid ceramidase OS=Homo sapiens GN=ASAH1
Eukaryotic translation initiation factor 5A-1 (Fragment) OS=Homo sapiens GN=EIF5A PE=1 SV=2
Eukaryotic translation initiation factor 5A-1 OS=Homo sapiens GN=EIF5A PE=1 SV=2
Isoform 2 of Eukaryotic translation initiation factor 5A-1 OS=Homo sapiens GN=EIF5A
Eukaryotic translation initiation factor 5A-1 OS=Homo sapiens GN=EIF5A PE=1 SV=1
Eukaryotic translation initiation factor 5A-2 OS=Homo sapiens GN=EIF5A2 PE=1 SV=1
Eukaryotic translation initiation factor 5A-2 OS=Homo sapiens GN=EIF5A2 PE=1 SV=1
Eukaryotic translation initiation factor 5A-2 (Fragment) OS=Homo sapiens GN=EIF5A2 PE=4 SV=1
Eukaryotic translation initiation factor 5A-2 OS=Homo sapiens GN=EIF5A2 PE=1 SV=3
Thioredoxin OS=Homo sapiens GN=TXN PE=1 SV=3
Serpin B9 OS=Homo sapiens GN=SERPINB9 PE=1 SV=1
Isoform 2C of Catenin delta-1 OS=Homo sapiens GN=CTNND1
Isoform 2 of Catenin delta-1 OS=Homo sapiens GN=CTNND1
Isoform 2AC of Catenin delta-1 OS=Homo sapiens GN=CTNND1
Isoform 2A of Catenin delta-1 OS=Homo sapiens GN=CTNND1
Isoform 2BC of Catenin delta-1 OS=Homo sapiens GN=CTNND1
Isoform 2B of Catenin delta-1 OS=Homo sapiens GN=CTNND1
Isoform 2AB of Catenin delta-1 OS=Homo sapiens GN=CTNND1
Isoform 2ABC of Catenin delta-1 OS=Homo sapiens GN=CTNND1
Isoform 1 of Catenin delta-1 OS=Homo sapiens GN=CTNND1
Isoform 1C of Catenin delta-1 OS=Homo sapiens GN=CTNND1
Isoform 1AC of Catenin delta-1 OS=Homo sapiens GN=CTNND1
Catenin delta-1 OS=Homo sapiens GN=CTNND1 PE=1 SV=2
Isoform 1A of Catenin delta-1 OS=Homo sapiens GN=CTNND1
Isoform 1B of Catenin delta-1 OS=Homo sapiens GN=CTNND1
Isoform 1BC of Catenin delta-1 OS=Homo sapiens GN=CTNND1
Isoform 1AB of Catenin delta-1 OS=Homo sapiens GN=CTNND1
Catenin delta-1 OS=Homo sapiens GN=CTNND1 PE=1 SV=1
Eukaryotic translation initiation factor 3 subunit A OS=Homo sapiens GN=EIF3A PE=1 SV=1
Eukaryotic translation initiation factor 3 subunit A OS=Homo sapiens GN=EIF3A PE=1 SV=1
Serpin B6 OS=Homo sapiens GN=SERPINB6 PE=1 SV=3
Small ubiquitin-related modifier 2 OS=Homo sapiens GN=SUMO2 PE=1 SV=3
Rab GDP dissociation inhibitor alpha OS=Homo sapiens GN=GDI1 PE=1 SV=2
Disintegrin and metalloproteinase domain-containing protein 10 OS=Homo sapiens GN=ADAM10 PE=1 SV=1
Inorganic pyrophosphatase OS=Homo sapiens GN=PPA1 PE=1 SV=2
Isoform 2 of ATP-citrate synthase OS=Homo sapiens GN=ACLY
ATP-citrate synthase OS=Homo sapiens GN=ACLY PE=1 SV=3
Crk-like protein OS=Homo sapiens GN=CRKL PE=1 SV=1
LIM domain containing preferred translocation partner in lipoma isoform CRA_e OS=Homo sapiens GN=LPP P
Lipoma-preferred partner OS=Homo sapiens GN=LPP PE=1 SV=1

Suppressor of tumorigenicity 14 protein OS=Homo sapiens GN=ST14 PE=1 SV=2
Lymphocyte antigen 75 OS=Homo sapiens GN=LY75 PE=1 SV=3
Isoform 3 of Lymphocyte antigen 75 OS=Homo sapiens GN=LY75
Isoform 2 of Lymphocyte antigen 75 OS=Homo sapiens GN=LY75
Cathepsin L1 OS=Homo sapiens GN=CTSL PE=1 SV=2
Angiotensinogen OS=Homo sapiens GN=AGT PE=1 SV=1
L-lactate dehydrogenase A chain OS=Homo sapiens GN=LDHA PE=1 SV=2
Isoform 3 of L-lactate dehydrogenase A chain OS=Homo sapiens GN=LDHA
Isoform 2 of Dihydropyrimidinase-related protein 2 OS=Homo sapiens GN=DPYSL2
Dihydropyrimidinase-related protein 2 OS=Homo sapiens GN=DPYSL2 PE=1 SV=1
Peroxiredoxin-2 OS=Homo sapiens GN=PRDX2 PE=1 SV=5
Tubulin beta chain OS=Homo sapiens GN=TUBB PE=1 SV=1
Tubulin beta chain OS=Homo sapiens GN=TUBB PE=1 SV=2
Tubulin beta chain OS=Homo sapiens GN=TUBB PE=1 SV=1
Stromal cell-derived factor 2-like protein 1 OS=Homo sapiens GN=SDF2L1 PE=1 SV=2
Aspartate aminotransferase cytoplasmic OS=Homo sapiens GN=GOT1 PE=1 SV=3
Aspartate aminotransferase OS=Homo sapiens GN=GOT1 PE=1 SV=1
Isoform 2 of Aminoacylase-1 OS=Homo sapiens GN=ACY1
Isoform 4 of Aminoacylase-1 OS=Homo sapiens GN=ACY1
Aminoacylase-1 OS=Homo sapiens GN=ACY1 PE=1 SV=1
Aminoacylase-1 OS=Homo sapiens GN=ACY1 PE=1 SV=1
Isoform 2 of Plasminogen activator inhibitor 1 RNA-binding protein OS=Homo sapiens GN=SERBP1
Plasminogen activator inhibitor 1 RNA-binding protein OS=Homo sapiens GN=SERBP1 PE=1 SV=2
Zyxin (Fragment) OS=Homo sapiens GN=ZYX PE=1 SV=1
Zyxin OS=Homo sapiens GN=ZYX PE=1 SV=1
Polymeric immunoglobulin receptor OS=Homo sapiens GN=PIGR PE=1 SV=4
Isoform 2 of Macrophage-capping protein OS=Homo sapiens GN=CAPG
Macrophage-capping protein OS=Homo sapiens GN=CAPG PE=1 SV=2
Staphylococcal nuclease domain-containing protein 1 OS=Homo sapiens GN=SND1 PE=1 SV=1
Isoform DeltaLf of Lactotransferrin OS=Homo sapiens GN=LTF
Kaliocin-1 (Fragment) OS=Homo sapiens GN=LTF PE=1 SV=1
Kaliocin-1 OS=Homo sapiens GN=LTF PE=1 SV=1
Lactotransferrin OS=Homo sapiens GN=LTF PE=1 SV=6
Isoform 3 of PC4 and SFRS1-interacting protein OS=Homo sapiens GN=PSIP1
Isoform 2 of PC4 and SFRS1-interacting protein OS=Homo sapiens GN=PSIP1
PC4 and SFRS1-interacting protein OS=Homo sapiens GN=PSIP1 PE=1 SV=1
PRKC apoptosis WT1 regulator protein OS=Homo sapiens GN=PAWR PE=1 SV=1
Kunitz-type protease inhibitor 1 (Fragment) OS=Homo sapiens GN=SPINT1 PE=1 SV=1
Isoform 2 of Kunitz-type protease inhibitor 1 OS=Homo sapiens GN=SPINT1
Kunitz-type protease inhibitor 1 OS=Homo sapiens GN=SPINT1 PE=1 SV=2
Calcium-regulated heat stable protein 1 OS=Homo sapiens GN=CARHSP1 PE=1 SV=2
Prostasin OS=Homo sapiens GN=PRSS8 PE=1 SV=1
Dipeptidyl peptidase 1 OS=Homo sapiens GN=CTSC PE=1 SV=2
SH3 domain-binding glutamic acid-rich-like protein 3 OS=Homo sapiens GN=SH3BGRL3 PE=1 SV=1
SH3 domain-binding glutamic acid-rich-like protein 3 OS=Homo sapiens GN=SH3BGRL3 PE=1 SV=1
Diphosphoinositol polyphosphate phosphohydrolase 1 OS=Homo sapiens GN=NUDT3 PE=1 SV=1
UMP-CMP kinase OS=Homo sapiens GN=CMPK1 PE=1 SV=1
UMP-CMP kinase OS=Homo sapiens GN=CMPK1 PE=1 SV=3
Isoform LCRMP-4 of Dihydropyrimidinase-related protein 3 OS=Homo sapiens GN=DPYSL3
Endoplasmic reticulum resident protein 29 OS=Homo sapiens GN=ERP29 PE=1 SV=4

Transthyretin OS=Homo sapiens GN=TTR PE=1 SV=1
LIM domain only protein 7 OS=Homo sapiens GN=LMO7 PE=1 SV=1
Nucleoprotein TPR OS=Homo sapiens GN=TPR PE=1 SV=3
F-actin-capping protein subunit alpha-1 OS=Homo sapiens GN=CAPZA1 PE=1 SV=3
Isoform Alpha of Nectin-2 OS=Homo sapiens GN=PVRL2
Nectin-2 OS=Homo sapiens GN=PVRL2 PE=1 SV=1
Ubiquitin-60S ribosomal protein L40 (Fragment) OS=Homo sapiens GN=UBA52 PE=4 SV=1
Ubiquitin (Fragment) OS=Homo sapiens GN=UBB PE=4 SV=1
Ubiquitin (Fragment) OS=Homo sapiens GN=RPS27A PE=1 SV=1
Ubiquitin-60S ribosomal protein L40 OS=Homo sapiens GN=UBA52 PE=1 SV=2
Ubiquitin-40S ribosomal protein S27a OS=Homo sapiens GN=RPS27A PE=1 SV=2
Polyubiquitin-C (Fragment) OS=Homo sapiens GN=UBC PE=1 SV=2
Ubiquitin (Fragment) OS=Homo sapiens GN=UBB PE=4 SV=1
Protein UBBP4 OS=Homo sapiens GN=UBBP4 PE=4 SV=1
Protein UBBP4 OS=Homo sapiens GN=UBBP4 PE=4 SV=1
Polyubiquitin-B OS=Homo sapiens GN=UBB PE=1 SV=1
Polyubiquitin-C OS=Homo sapiens GN=UBC PE=2 SV=1
Polyubiquitin-C OS=Homo sapiens GN=UBC PE=1 SV=3
Beta-hexosaminidase subunit beta OS=Homo sapiens GN=HEXB PE=1 SV=3
Isoform 2 of Sortilin OS=Homo sapiens GN=SORT1
Sortilin OS=Homo sapiens GN=SORT1 PE=1 SV=3
Thyrotropin-releasing hormone-degrading ectoenzyme OS=Homo sapiens GN=TRHDE PE=2 SV=1
Biotinidase OS=Homo sapiens GN=BTD PE=4 SV=1
Isoform 2 of Biotinidase OS=Homo sapiens GN=BTD
Isoform 3 of Biotinidase OS=Homo sapiens GN=BTD
Biotinidase OS=Homo sapiens GN=BTD PE=1 SV=2
Tubulin beta-4B chain OS=Homo sapiens GN=TUBB4B PE=1 SV=1
Isoform 4 of Heterogeneous nuclear ribonucleoprotein H3 OS=Homo sapiens GN=HNRNPH3
Heterogeneous nuclear ribonucleoprotein H3 OS=Homo sapiens GN=HNRNPH3 PE=1 SV=1
Isoform 3 of Heterogeneous nuclear ribonucleoprotein H3 OS=Homo sapiens GN=HNRNPH3
Isoform 2 of Heterogeneous nuclear ribonucleoprotein H3 OS=Homo sapiens GN=HNRNPH3
Heterogeneous nuclear ribonucleoprotein H3 OS=Homo sapiens GN=HNRNPH3 PE=1 SV=2
Activated RNA polymerase II transcriptional coactivator p15 OS=Homo sapiens GN=SUB1 PE=1 SV=3
Transaldolase OS=Homo sapiens GN=TALDO1 PE=1 SV=2
Follistatin-related protein 1 OS=Homo sapiens GN=FSTL1 PE=1 SV=1
Adenylosuccinate synthetase isozyme 2 OS=Homo sapiens GN=ADSS PE=1 SV=3
Isoform 4 of 45 kDa calcium-binding protein OS=Homo sapiens GN=SDF4
45 kDa calcium-binding protein OS=Homo sapiens GN=SDF4 PE=1 SV=1
Isoform 3 of 45 kDa calcium-binding protein OS=Homo sapiens GN=SDF4
Isoform 6 of 45 kDa calcium-binding protein OS=Homo sapiens GN=SDF4
45 kDa calcium-binding protein OS=Homo sapiens GN=SDF4 PE=1 SV=1
Phosphoribosylaminoimidazole carboxylase (Fragment) OS=Homo sapiens GN=PAICS PE=1 SV=1
Multifunctional protein ADE2 OS=Homo sapiens GN=PAICS PE=1 SV=3
Isoform 2 of Multifunctional protein ADE2 OS=Homo sapiens GN=PAICS
60 kDa heat shock protein mitochondrial OS=Homo sapiens GN=HSPD1 PE=1 SV=2
Alpha-N-acetylgalactosaminidase OS=Homo sapiens GN=NAGA PE=1 SV=2
Glutamate dehydrogenase 1 mitochondrial OS=Homo sapiens GN=GLUD1 PE=1 SV=2
Delta(3 5)-Delta(2 4)-dienoyl-CoA isomerase mitochondrial OS=Homo sapiens GN=ECH1 PE=1 SV=2
Actin-related protein 2 OS=Homo sapiens GN=ACTR2 PE=1 SV=1
Isoform 2 of V-set domain-containing T-cell activation inhibitor 1 OS=Homo sapiens GN=VTCN1

Isoform 4 of V-set domain-containing T-cell activation inhibitor 1 OS=Homo sapiens GN=VTCN1
V-set domain-containing T-cell activation inhibitor 1 OS=Homo sapiens GN=VTCN1 PE=4 SV=1
V-set domain-containing T-cell activation inhibitor 1 OS=Homo sapiens GN=VTCN1 PE=1 SV=1
Glycylpeptide N-tetradecanoyltransferase 1 OS=Homo sapiens GN=NMT1 PE=1 SV=1
Glycylpeptide N-tetradecanoyltransferase 1 OS=Homo sapiens GN=NMT1 PE=1 SV=2
Mammalian ependymin-related protein 1 OS=Homo sapiens GN=EPDR1 PE=1 SV=2
Ependymin related protein 1 (Zebrafish) isoform CRA_b OS=Homo sapiens GN=UCC1 PE=4 SV=1
Isoform DSPIa of Desmoplakin OS=Homo sapiens GN=DSP
Desmoplakin OS=Homo sapiens GN=DSP PE=1 SV=3
Matrin-3 (Fragment) OS=Homo sapiens GN=MATR3 PE=1 SV=1
Matrin-3 OS=Homo sapiens GN=MATR3 PE=1 SV=1
Matrin-3 OS=Homo sapiens GN=MATR3 PE=1 SV=2
Matrin-3 OS=Homo sapiens GN=MATR3 PE=1 SV=1
Alpha-fetoprotein OS=Homo sapiens GN=AFP PE=1 SV=1
Alpha-fetoprotein OS=Homo sapiens GN=AFP PE=1 SV=1
Protein ERGIC-53 OS=Homo sapiens GN=LMAN1 PE=1 SV=2
Neudesin OS=Homo sapiens GN=NENF PE=1 SV=1
Splicing factor proline- and glutamine-rich OS=Homo sapiens GN=SFPQ PE=1 SV=2
Serine/arginine-rich splicing factor 9 OS=Homo sapiens GN=SRSF9 PE=1 SV=1
Neutrophil gelatinase-associated lipocalin OS=Homo sapiens GN=LCN2 PE=1 SV=2
Isoform 2 of Neutrophil gelatinase-associated lipocalin OS=Homo sapiens GN=LCN2
Neutrophil gelatinase-associated lipocalin OS=Homo sapiens GN=LCN2 PE=3 SV=1
Neutrophil gelatinase-associated lipocalin OS=Homo sapiens GN=LCN2 PE=1 SV=1
TIP41-like protein OS=Homo sapiens GN=TIPRL PE=1 SV=2
Isoform 17 of Fibronectin OS=Homo sapiens GN=FN1
Isoform 4 of Interleukin-1 receptor accessory protein OS=Homo sapiens GN=IL1RAP
Isoform 2 of Interleukin-1 receptor accessory protein OS=Homo sapiens GN=IL1RAP
Interleukin-1 receptor accessory protein OS=Homo sapiens GN=IL1RAP PE=1 SV=2
Elongation factor 1-gamma OS=Homo sapiens GN=EEF1G PE=1 SV=3
Elongation factor 1-gamma OS=Homo sapiens GN=EEF1G PE=1 SV=1
Purine nucleoside phosphorylase OS=Homo sapiens GN=PNP PE=1 SV=2
Annexin A2 OS=Homo sapiens GN=ANXA2 PE=1 SV=2
Isoform 2 of Annexin A2 OS=Homo sapiens GN=ANXA2
Thrombospondin-1 OS=Homo sapiens GN=THBS1 PE=1 SV=2
Histidine--tRNA ligase cytoplasmic (Fragment) OS=Homo sapiens GN=HARS PE=1 SV=1
Histidine--tRNA ligase cytoplasmic OS=Homo sapiens GN=HARS PE=1 SV=1
Isoform 4 of Histidine--tRNA ligase cytoplasmic OS=Homo sapiens GN=HARS
Histidine--tRNA ligase cytoplasmic OS=Homo sapiens GN=HARS PE=1 SV=2
Histidine--tRNA ligase cytoplasmic OS=Homo sapiens GN=HARS PE=1 SV=1
Isoform 3 of Histidine--tRNA ligase cytoplasmic OS=Homo sapiens GN=HARS
Isoform 2 of Histidine--tRNA ligase cytoplasmic OS=Homo sapiens GN=HARS
Isoform 5 of EGF-containing fibulin-like extracellular matrix protein 1 OS=Homo sapiens GN=EFEMP1
Isoform 2 of EGF-containing fibulin-like extracellular matrix protein 1 OS=Homo sapiens GN=EFEMP1
Isoform 4 of EGF-containing fibulin-like extracellular matrix protein 1 OS=Homo sapiens GN=EFEMP1
Isoform 3 of EGF-containing fibulin-like extracellular matrix protein 1 OS=Homo sapiens GN=EFEMP1
EGF-containing fibulin-like extracellular matrix protein 1 OS=Homo sapiens GN=EFEMP1 PE=1 SV=2
Antithrombin-III OS=Homo sapiens GN=SERPINC1 PE=1 SV=1
Transferrin receptor (P90 CD71) isoform CRA_c OS=Homo sapiens GN=TFRC PE=1 SV=1
Transferrin receptor protein 1 OS=Homo sapiens GN=TFRC PE=1 SV=2
Inter-alpha-trypsin inhibitor heavy chain H2 OS=Homo sapiens GN=ITIH2 PE=1 SV=1

Inter-alpha-trypsin inhibitor heavy chain H2 OS=Homo sapiens GN=ITIH2 PE=1 SV=2
Isoform TGN46 of Trans-Golgi network integral membrane protein 2 OS=Homo sapiens GN=TGOLN2
Isoform 5 of Trans-Golgi network integral membrane protein 2 OS=Homo sapiens GN=TGOLN2
Trans-Golgi network integral membrane protein 2 OS=Homo sapiens GN=TGOLN2 PE=1 SV=1
Isoform TGN48 of Trans-Golgi network integral membrane protein 2 OS=Homo sapiens GN=TGOLN2
Trans-Golgi network integral membrane protein 2 OS=Homo sapiens GN=TGOLN2 PE=1 SV=2
NAD(P)H-hydrate epimerase OS=Homo sapiens GN=APOA1BP PE=1 SV=2
Hepatoma-derived growth factor-related protein 3 OS=Homo sapiens GN=HDGFRP3 PE=1 SV=1
Isoform 2 of Heme-binding protein 2 OS=Homo sapiens GN=HEBP2
Heme-binding protein 2 OS=Homo sapiens GN=HEBP2 PE=1 SV=1
Cadherin-1 OS=Homo sapiens GN=CDH1 PE=1 SV=1
Cadherin-1 OS=Homo sapiens GN=CDH1 PE=1 SV=1
Cadherin 1 type 1 E-cadherin (Epithelial) isoform CRA_c OS=Homo sapiens GN=CDH1 PE=1 SV=1
Cadherin-1 OS=Homo sapiens GN=CDH1 PE=1 SV=3
Hepatoma-derived growth factor OS=Homo sapiens GN=HDGF PE=1 SV=1
Golgi apparatus protein 1 OS=Homo sapiens GN=GLG1 PE=1 SV=1
Golgi apparatus protein 1 OS=Homo sapiens GN=GLG1 PE=1 SV=2
Isoform 3 of Golgi apparatus protein 1 OS=Homo sapiens GN=GLG1
Isoform 2 of Golgi apparatus protein 1 OS=Homo sapiens GN=GLG1
Di-N-acetylchitobiase OS=Homo sapiens GN=CTBS PE=1 SV=1
Annexin OS=Homo sapiens GN=ANXA5 PE=1 SV=1
Annexin A5 OS=Homo sapiens GN=ANXA5 PE=1 SV=2
Annexin A1 OS=Homo sapiens GN=ANXA1 PE=1 SV=2
Hypoxanthine-guanine phosphoribosyltransferase OS=Homo sapiens GN=HPRT1 PE=1 SV=2
Protein disulfide-isomerase A3 OS=Homo sapiens GN=PDIA3 PE=1 SV=4
Eukaryotic translation initiation factor 4B OS=Homo sapiens GN=EIF4B PE=1 SV=1
Eukaryotic translation initiation factor 4B OS=Homo sapiens GN=EIF4B PE=1 SV=2
Eukaryotic translation initiation factor 4B OS=Homo sapiens GN=EIF4B PE=1 SV=1
Semaphorin-4B (Fragment) OS=Homo sapiens GN=SEMA4B PE=4 SV=1
Semaphorin-4B (Fragment) OS=Homo sapiens GN=SEMA4B PE=4 SV=1
Isoform 2 of Semaphorin-4B OS=Homo sapiens GN=SEMA4B
Semaphorin-4B OS=Homo sapiens GN=SEMA4B PE=1 SV=3
Semaphorin-4B OS=Homo sapiens GN=SEMA4B PE=1 SV=1
Isoform 2 of 182 kDa tankyrase-1-binding protein OS=Homo sapiens GN=TNKS1BP1
182 kDa tankyrase-1-binding protein OS=Homo sapiens GN=TNKS1BP1 PE=1 SV=4
Alpha-2-macroglobulin OS=Homo sapiens GN=A2M PE=1 SV=3
Ubiquilin-2 OS=Homo sapiens GN=UBQLN2 PE=1 SV=2
Isoform 2 of Ubiquilin-1 OS=Homo sapiens GN=UBQLN1
Ubiquilin-1 OS=Homo sapiens GN=UBQLN1 PE=1 SV=2
Receptor-type tyrosine-protein phosphatase beta (Fragment) OS=Homo sapiens GN=PTPRB PE=1 SV=1
Isoform 4 of Receptor-type tyrosine-protein phosphatase beta OS=Homo sapiens GN=PTPRB
Receptor-type tyrosine-protein phosphatase beta OS=Homo sapiens GN=PTPRB PE=1 SV=1
Receptor-type tyrosine-protein phosphatase beta OS=Homo sapiens GN=PTPRB PE=1 SV=3
Isoform 3 of Receptor-type tyrosine-protein phosphatase beta OS=Homo sapiens GN=PTPRB
Receptor-type tyrosine-protein phosphatase beta OS=Homo sapiens GN=PTPRB PE=1 SV=1
Isoform 2 of Receptor-type tyrosine-protein phosphatase beta OS=Homo sapiens GN=PTPRB
Isoform Short of Beta-1 4-galactosyltransferase 1 OS=Homo sapiens GN=B4GALT1
Beta-1 4-galactosyltransferase 1 OS=Homo sapiens GN=B4GALT1 PE=1 SV=5
Septin-11 (Fragment) OS=Homo sapiens GN=SEPT11 PE=1 SV=1
Septin 11 isoform CRA_b OS=Homo sapiens GN=SEPT11 PE=1 SV=1

Septin-11 OS=Homo sapiens GN=SEPT11 PE=1 SV=3
Septin-11 OS=Homo sapiens GN=SEPT11 PE=1 SV=1
Isoform 2 of Septin-11 OS=Homo sapiens GN=SEPT11
Dickkopf-related protein 1 OS=Homo sapiens GN=DKK1 PE=1 SV=1
28 kDa heat- and acid-stable phosphoprotein OS=Homo sapiens GN=PDAP1 PE=1 SV=1
LDLR chaperone MESD OS=Homo sapiens GN=MESDC2 PE=1 SV=2
Isocitrate dehydrogenase [NADP] cytoplasmic OS=Homo sapiens GN=IDH1 PE=1 SV=2
Dipeptidyl peptidase 2 OS=Homo sapiens GN=DPP7 PE=1 SV=3
Ephrin-B1 OS=Homo sapiens GN=EFNB1 PE=1 SV=1
Leucine-rich repeat transmembrane protein FLRT3 OS=Homo sapiens GN=FLRT3 PE=1 SV=1
GDP-L-fucose synthase (Fragment) OS=Homo sapiens GN=TSTA3 PE=1 SV=1
GDP-L-fucose synthase (Fragment) OS=Homo sapiens GN=TSTA3 PE=1 SV=1
GDP-L-fucose synthase OS=Homo sapiens GN=TSTA3 PE=1 SV=1
Phospholipase D3 OS=Homo sapiens GN=PLD3 PE=1 SV=1
Lysine--tRNA ligase OS=Homo sapiens GN=KARS PE=1 SV=3
Isoform Mitochondrial of Lysine--tRNA ligase OS=Homo sapiens GN=KARS
Melanotransferrin OS=Homo sapiens GN=MFI2 PE=1 SV=2
Calcyphosin OS=Homo sapiens GN=CAPS PE=1 SV=1
Calcyphosin OS=Homo sapiens GN=CAPS PE=4 SV=1
Isoform 2 of Pyridoxal kinase OS=Homo sapiens GN=PDXK
Pyridoxal kinase OS=Homo sapiens GN=PDXK PE=1 SV=1
Chromobox protein homolog 3 (Fragment) OS=Homo sapiens GN=CBX3 PE=1 SV=1
Chromobox homolog 3 (HP1 gamma homolog Drosophila) isoform CRA_b OS=Homo sapiens GN=CBX3 PE=1 [illegible]
Chromobox protein homolog 3 (Fragment) OS=Homo sapiens GN=CBX3 PE=1 SV=1
Chromobox protein homolog 3 OS=Homo sapiens GN=CBX3 PE=1 SV=4
Dermcidin OS=Homo sapiens GN=DCD PE=1 SV=2
Isoform 2 of Dermcidin OS=Homo sapiens GN=DCD
Leucine-rich alpha-2-glycoprotein OS=Homo sapiens GN=LRG1 PE=1 SV=2
Kallistatin OS=Homo sapiens GN=SERPINA4 PE=1 SV=3
S-formylglutathione hydrolase (Fragment) OS=Homo sapiens GN=ESD PE=1 SV=1
S-formylglutathione hydrolase OS=Homo sapiens GN=ESD PE=4 SV=1
S-formylglutathione hydrolase OS=Homo sapiens GN=ESD PE=1 SV=2
Isoform K of Membrane cofactor protein OS=Homo sapiens GN=CD46
Isoform N of Membrane cofactor protein OS=Homo sapiens GN=CD46
Isoform L of Membrane cofactor protein OS=Homo sapiens GN=CD46
Isoform E of Membrane cofactor protein OS=Homo sapiens GN=CD46
Isoform I of Membrane cofactor protein OS=Homo sapiens GN=CD46
Isoform M of Membrane cofactor protein OS=Homo sapiens GN=CD46
Isoform F of Membrane cofactor protein OS=Homo sapiens GN=CD46
Isoform J of Membrane cofactor protein OS=Homo sapiens GN=CD46
Isoform C of Membrane cofactor protein OS=Homo sapiens GN=CD46
Isoform G of Membrane cofactor protein OS=Homo sapiens GN=CD46
Isoform D of Membrane cofactor protein OS=Homo sapiens GN=CD46
Isoform H of Membrane cofactor protein OS=Homo sapiens GN=CD46
Isoform 2 of Membrane cofactor protein OS=Homo sapiens GN=CD46
Membrane cofactor protein OS=Homo sapiens GN=CD46 PE=1 SV=3
Isoform B of Membrane cofactor protein OS=Homo sapiens GN=CD46

:ens GN=SLC12A2 PE=1 SV=1

;V=4

V=1

/=1

E=1 SV=1

SV=1
