# Supplementary material for: Neisseria Heparin Binding Antigen is targeted by the human alternative pathway C3-convertase
Source: PLoS One. 2018 Mar 26;13(3):e0194662. doi: 10.1371/journal.pone.0194662 (PMC5868813; doi:10.1371/journal.pone.0194662)
Supplement: S1 Text — (DOCX) [file pone.0194662.s009.docx]

**Expression and purification of recombinant proteins**

Recombinant NHBA full-length protein or C2 fragment (**7**) was expressed in *E. coli* BL21 (DE3) strain by using EnPresso B growth kit (BioSilta) supplemented with 100 μg/ml ampicillin. Bacteria were grown at 30°C for 16 h, and protein expression was induced by the addition of 1 mM isopropyl β-d-1-thiogalactopyranoside (IPTG) (Sigma) at 25°C for 24 h. The soluble proteins were extracted by sonication in 50 mM NaH_2_PO_4_, 300 mM NaCl, 10 mM imidazole, pH=8, supplemented with protease inhibitors (cOmplete EDTA-free Protease Inhibitor Cocktail; Roche), followed by centrifugation to remove cell debris. Recombinant proteins were purified from supernatant by affinity chromatography with HisTrap FF Crude column (GE Healthcare) using a peristaltic pump (flow rate 0.5 mL/minute). After a washing step with 50mM imidazole to remove *E. coli* contaminants, recombinant proteins were eluted at a concentration of 250 mM imidazole. Eluted sample was dialyzed at 4 °C overnight against 10 mM NaH_2_PO_4_, pH=7, buffer using Snake Skin Dialysis Tubing, 10,000 MWCO, 22 mm (Thermo Scientific), and a second step of purification was performed to obtain a high pure product. Dialyzed sample was load on 1 mL HiTrap heparin HP column (GE Healthcare) using a peristaltic pump (flow rate 0.5 mL/minute). After a washing step with 30 mM NaCl, recombinant proteins were eluted at a concentration of 350 mM NaCl. PD-10 Desalting column (GE Healthcare) was used for buffer exchange and proteins were eluted in PBS. Protein content was quantified using the BCA Kit (Themo Fischer Scientific). Purity was checked by SDS-PAGE analysis, as well as by SEC-UPLC loading the sample on BEH200 4,6x300mm column (Waters). Size exclusion chromatography was performed using 10 mM NaH_2_PO_4_, 400 mM (NH_4_)_3_PO_4_, pH=6, buffer. Endotoxin content was quantified by LAL assay using ENDOSAFE instrument (Charles River).

**BSA-FITC/ Dextran-Texas Red permeability assay**

The formation of intact monolayers on transwell inserts was evaluated by adding BSA- FITC (Thermo Scientific) or 10,000 MW Dextran-Texas Red (Thermo Scientific) at the concentration of 1 mg/mL to the upper chamber and measuring after 1 h the amount of labeled probe passed into the lower chamber by a fluorescence microplate reader (TECAN). Transwell inserts were used only when the intensity of fluorescence in the lower chamber was negligible. 5 µM of recombinant C2 fragment or PBS, as negative control, were added to the upper chamber of selected transwell inserts, and the fluorescence was evaluated in the lower chamber at various time intervals (45 min, 2 h, 4 h, and 24 h). The permeability of cell monolayers for a probe was quantified calculating the ∆ mean of fluorescent intensity normalized versus pre-treatment values.

**Immunofluorescence analysis**

Cell monolayers were washed with PBS and fixed for 20 min in 2 % (v/v) formaldehyde (Carlo Erba Reagents) in PBS. After several washing steps using PBS, cell monolayers were removed from transwell inserts by cutting the membranes. Samples were permeabilized with 0.1 % (v/v) Triton X-100 in PBS for 10 min and blocked for 10 min with 10 % (v/v) of goat serum (Invitrogen). Incubation with the primary rabbit anti-ZO 1 antibody (working dilution 1: 200, REF. 402200, Lot. QA213066; Life Technologies) was performed for 1 h, at RT, under gently agitation. After several washing steps, samples were incubated for 45 min, at RT, under gently agitation, with Alexa flour 568-conjugated goat anti rabbit IgG secondary antibody (working dilution 1: 1,000, Life Technologies). All washing steps and antibody dilutions were performed using 0.01 % (v/v)Triton X-100, 3 % (w/v) bovine serum albumin (Sigma) in PBS. Lastly, after washing, labeled preparations were mounted using prolong gold antifade reagent with DAPI (Molecular probes-Life Technologies) and analyzed with a confocal microscope (Zeiss). Cell nuclei and tight junction structures were examined using the UV and red filter sets, respectively.

**RNA extraction and semi-quantitative PCR**

Calu-3 and polarized Calu-3 cells were used for RNA isolation. Total RNA was extracted using the TRIzol reagent (Ambion) according to the manufacturer’s instructions, precipitated and resuspended in RNase free water (Ambion). The yields of total RNA obtained were quantified against water at 260 nm using Nanodrop spectropotometer. cDNA was prepared from 2 µg of total RNA by using GoScrip Reverse transcription system with oligo(dT) primers (Promega) according to the manufacturer’s instruction. The presence of cDNA for GADPH, Factor B and C3 were determined by PCR amplification. After an initial denaturation step at 94 °C for 5 min, temperature cycling was initiated. Each cycle consisted of 30 s at 98 °C, 30 s at 59 °C and 1 min at 72 °C; in total 35 cycles were performed. Primers used for PCR amplification are listed in the table below. Amplification products were separated by electrophoresis using 1.5 % (w/v) agarose gels, and stained with SYBR Safe DNA Gel Stain (Themo Fischer Scientific) in order to be visualized.

**Plasmids and primers used in this study**

| **Plasmids** | | | |
| --- | --- | --- | --- |
| **Strain** | **Carried plasmid** | **Relevant characteristics** | **Reference** |
| **BL21 (DE3)** | pET-GNA2132-MC58-his | pET21b derivative for expression of recombinant GNA2132 protein (MC58 strain - rGNA2132MC58-his) | (**7**) |
| **BL21 (DE3)** | pET-GNA2132-C-his | pET21b derivative for expression of recombinant C-terminal region (293-488) of GNA2132 protein (MC58 strain - C-his) | (**7**) |

| **Primers** | |
| --- | --- |
| **Primer ID** | **Primer sequence** |
| GAPDH forward | 5’- TCGGAGTCAACGGATTTGGTCG -3’ |
| GAPDH reverse | 5‘- GACTGTGGTCATGAGTCCTTCCA-3’ |
| Factor B forward | 5′- CAACAGAAGCGGAAGATCGTC -3′ |
| Factor B reverse | 5′- TATCTCCAGGTCCCGCTTCTC -3′ |
| C3 forward | 5’- TCGGATGACAAGGTCACCCT-3’ |
| C3 reverse | 5’- GACAACCATGCTCTCGGTGA -3’ |
